# Physician-physician handover from acute care to rehabilitation setting: A scoping review protocol

**DOI:** 10.1371/journal.pone.0338916

**Published:** 2025-12-12

**Authors:** Seungjae Cho, Nancy Xi, Emma A. Bateman, Cynthia Chui, Eric Poon, Aran Bains, Patrick Fangping Yao, Meiqi Guo

**Affiliations:** 1 Temerty Faculty of Medicine, University of Toronto, Toronto, Ontario, Canada; 2 Toronto Rehab Institute, University Health Network, Toronto, Ontario, Canada; 3 St Joseph’s Health Care London, London, Ontario, Canada; 4 Department of Physical Medicine & Rehabilitation, Schulich School of Medicine & Dentistry, University of Western Ontario, London, Ontario, Canada; 5 University Health Network, Toronto, Ontario, Canada; 6 Division of Physical Medicine & Rehabilitation, Department of Medicine, University of Toronto, Toronto, Ontario, Canada; 7 Schulich School of Medicine & Dentistry, University of Western Ontario, London, Ontario, Canada; 8 Department of Physical Medicine & Rehabilitation, McMaster University, Hamilton, Ontario, Canada; 9 Toronto Rehabilitation Institute-University Health Network, Toronto, Ontario, Canada; MountainView Hospital, UNITED STATES OF AMERICA

## Abstract

**Objective:**

The purpose of this scoping review is to map the existing evidence that describes strategies to improve handover from the acute care to rehabilitation settings.

**Introduction:**

Poor handover processes have been associated with preventable errors, delays in care, and adverse patient outcomes. Effective physician-to-physician handover during transitions of care is critical to ensuring patient safety and optimizing clinical outcomes. Physician handover between acute and rehabilitation care settings is particularly complicated, as it requires transferring detailed and timely information for continuity of care for medically and/or surgically complex patients between components of healthcare systems with different cultures and goals of care. Despite numerous studies being published on handover, there has yet to be a synthesis of the existing literature that seeks to explore handovers across acute to rehabilitation settings as well as how care transitions can be improved. This scoping review aims to map the existing evidence on physician-to-physician handover from acute care to rehabilitation.

**Methods:**

This review will be conducted following the Joanna Briggs Institute (JBI) framework and the Preferred Reporting Items for Systematic Reviews and Meta-Analyses Extension for Scoping Reviews (PRISMA-ScR) guidelines. A comprehensive search will be performed across the following electronic databases: MEDLINE(R) ALL (Ovid), Embase Classic + Embase (Ovid), APA PsycINFO (Ovid), Cochrane Central Register of Controlled Trials (Ovid), Emcare (Ovid), CINAHL Ultimate (EBSCO) and Web of Science (Clarivate). All rounds of screening, data extraction, and data synthesis will be conducted independently with each stage performed in duplicate. The extracted data will be summarized both quantitively with descriptive statistics and qualitatively using content analysis.

**Eligibility Criteria:**

Qualitative and quantitative studies published in English that discuss physician-physician handover from acute care to rehabilitation settings will be included. All geographical areas will be considered. Case reports, case series, commentaries, protocols, opinion pieces (editorials), or abstracts from conferences will be excluded.

## Introduction

Handover or handoff in a clinical setting refers to the transfer of responsibility and accountability of a patient’s care from one healthcare provider to another or from one care team to another. Effective handover is a critical component of patient care to ensure continuity, safety, and the minimization of errors during transitions between healthcare providers and settings [1]. Poor handover has been associated with adverse events, delayed diagnoses, and compromised patient outcomes [2]. According to Starmer et al., implementing a standardized handover program in pediatric hospitals reduced medical errors and preventable adverse events [3]. In the acute care setting, interventions have been developed to improve handovers such as the I-PASS handover bundle that incorporates mnemonic tools, dedicated training, and electronic documentation strategies. These approaches have demonstrated improvements in information transfer [4]. Collectively, the existing handover literature highlights that enhancing handover processes in acute care settings not only improves patient safety but also lays the foundation for successful transitions to subsequent phases of care such as rehabilitation.

Handover between acute care and rehabilitation settings is particularly complicated as it requires transferring detailed and timely information on medically and/or surgically complex patients to facilitate continuity of care between two components of the healthcare system with different cultures and goals of care. This transition occurs between two often fundamentally different phases of a patient’s health care journey, from acute care—focused on stabilization and life-saving interventions—to rehabilitation, which emphasizes post-stabilization functional improvement and reintegration into daily life. Despite its importance, the current literature regarding improving handover for the transition of care from acute to post-acute settings is limited. Consequently, there is often a communication gap between physicians in acute settings and physicians in post-acute rehabilitation settings such as skilled nursing facilities [5,6]. Such literature findings highlight the need to standardize and improve handover processes between physicians in acute and post-acute settings to ensure patient safety and continuity.

Given the complexity of handover from acute care to rehabilitation, there are many strategies employed at various points of the process that have been explored, aiming to optimize outcomes. Hill et al. highlighted the significance of structured communication in reducing errors during transitions for persons with stroke, including the potential for improved rehabilitation outcomes when handovers are clear and complete [7]. Similarly, Neufeld et al. demonstrated how a Lean/Six Sigma approach to enhance discharge documentation improved the quality of admissions to inpatient rehabilitation, underscoring the importance of thorough and standardized handover practices [8]. While individual studies have identified challenges and evaluated potential solutions on effective handovers, there has yet to be a comprehensive synthesis of the existing literature that explores how acute care to rehabilitation setting handovers can be optimized to improve patient care. Therefore, this scoping review aims to map the existing evidence on physician-to-physician handover from acute care to rehabilitation.

### Review question

What strategies can improve physician-to-physician handover from acute care to rehabilitation?

## Methods

This protocol will follow the Joanna Briggs Institute (JBI) methodological framework for scoping reviews. The JBI framework provides guidance on structuring the review, defining inclusion criteria using the Population, Concept, and Context (PCC) framework, developing a search strategy, extracting data, presenting and summarizing results, and discussing potential implications [9]. The scoping review will be reported using the Preferred Reporting Items for Systematic Reviews and Meta-Analyses Extension for Scoping Reviews (PRISMA-ScR) guidelines [10]. This ensures the completion of all essential components of a high-quality scoping review.

### Protocol and registration

The protocol was registered and published on Open Science Framework (Registration https://doi.org/10.17605/OSF.IO/Y6DRU).

### Eligibility criteria

The selection of studies will be guided by specific inclusion and exclusion criteria to ensure the relevance and focus of this scoping review. All study designs written in English, both qualitative and quantitative studies, will be considered for inclusion. Although studies will initially be included during title and abstract screening stage if abstracts are written in English, they will be excluded if full-text studies are not available in English. All geographical areas will be considered. This broad approach allows for a comprehensive understanding of the strategies employed in physician-to-physician handovers.

The review will focus on studies that discuss physician-physician handovers for patients being transferred from acute care to rehabilitation settings. Studies involving patients of all ages in handovers will be eligible for inclusion. Acute care will be defined broadly to encompass a wide range of medical and surgical services typically involved in the initial management of patients prior to rehabilitation. Some examples of services include general internal medicine, surgery, cardiology, oncology, intensive care and emergency units. Freestanding rehabilitation hospitals, non-freestanding rehabilitation and skilled nursing facilities were considered as rehabilitation services. By including diverse post-acute care settings, the review aims to capture a wide range of handover practices and strategies.

For the purpose of this scoping review, “physician” will be defined as any individual holding a Doctor of Medicine (MD) degree or equivalent, including but not limited to physiatrists, hospitalists, residents or other medical doctors involved in patient care. Studies describing multi-disciplinary handover processes with other health professionals will also be included if a physician was either providing or receiving handover. Handovers within a rehabilitation hospital or handovers between nursing staff or other allied health professionals will be excluded.

Case reports, case series, commentaries, protocols, opinion pieces (editorials), or abstracts from conferences will be excluded.

### Search strategy

A comprehensive search strategy was developed in consultation with a health sciences librarian experienced in systematic and scoping reviews. The search was conducted across the following electronic databases: MEDLINE(R) ALL (Ovid), Embase Classic + Embase (Ovid), APA PsycINFO (Ovid), Cohrane Central Register of Controlled Trials (Ovid), Emcare (Ovid), CINAHL Ultimate (EBSCO), Web of Science Core Collection (Clarivate). The initial search strategy was developed in MEDLINE(R) ALL and tested using a set of target articles provided by the team.

The search strategy included subject headings (e.g., MeSH) and text words for 4 search concepts: A) Inpatient rehab facilities, B) handoff/handover, and C) care transitions, and D) communication/information exchange. Our final search strategy is A AND (B OR (C AND D)) which translates to 1) Inpatient rehab facilities AND handoff/handover, and 2) Inpatient rehab facilities AND care transitions AND communication/information exchange.

“Acute care” as a separate search concept was not included in our search strategy to avoid unintentionally excluding relevant studies. Defining and capturing the full scope of acute care settings using database search terms is highly complex as “acute care” may encompass a wide range of medical and surgical specialties such as general medicine, surgery, cardiology, oncology, intensive care, and emergency services. Therefore, including “acute care” search terms could have inadvertently filtered out eligible studies describing transitions to rehabilitation. Instead, we focused the search on rehabilitation-related settings, which inherently implies a transition from an acute care environment. This approach aligns with the aim of the scoping review which is to examine physician-to-physician handovers occurring at the point of transfer from acute care to rehabilitation.

Some key search terms included: rehabilitation hospitals, inpatient rehabilitation, handoff, handover, signovers, etc. No limits were applied to the search strategy (e.g., publication date, language, etc.). The MEDLINE(R) ALL search strategy was later revised with recommendations from the PRESS (Peer Review of Electronic Search Strategies) by a medical librarian. We thank Emilia Main, MI (Toronto Rehab, University Health Network) for peer review of the submitted search strategy. The final Medline strategy was then translated to the other electronic databases. For the full search strategies, please refer to S1 Appendix. The databases were searched from inception to February 5, 2025, and will be updated prior to submission of the scoping review for publication if more than 6 months has elapsed since the last search date. Citation tracking will also be used as a supplementary search method. The reference lists of the included studies will be reviewed for additional studies that meet the inclusion and exclusion criteria. Web of Science will use used to generate a citation report for forward and backward citation tracking of included studies.

### Study/Source of evidence selection

Following database searches, all identified studies will be uploaded to Covidence. Following a pilot test to ensure consistent application of inclusion criteria, each study will undergo title and abstract screening by two independent reviewers. Full-text articles that meet the inclusion criteria from title and abstract screening will be retrieved. A subsequent round of screening will be conducted independently by two reviewers on the full-text articles. Each round will be screened in duplicate. Screening agreement will be reached when there are two yes or two no votes in Covidence. If any conflicts arise, an additional screener will resolve the disagreement. The voting decisions will be blinded to the additional screener. A PRISMA-ScR flow diagram will visually present the entire study screening process. For the full PRISMA-ScR flow diagram, please refer to S2 Appendix.

### Data extraction

The data extraction process will consist of a pilot stage followed by final extraction. They will be completed in duplicate by four extractors and discrepancies in data extraction will be resolved by an additional extractor. We will employ the Template for Intervention Description and Replication (TIDieR) template to ensure sufficient detail extraction regarding the interventions and any additional entities relevant to the concepts in this study [11]. Upon reasonable request, the CSV data will be made available. Data collection will be expected to be completed by Sept 1^st^, 2025.

The extracted entities will include: study title, country of origin, year of publication, and study design (e.g., qualitative, quasi-experimental, randomized controlled trial). In accordance with the TIDieR framework, we will extract the rationale (describing the theory or goal of the intervention’s key elements), what was done to increase handover (e.g., department education session followed by policy implementation), materials used (e.g., “I-PASS” lanyards, EMR handover template), procedures (detailed description of intervention activities and processes), the profession of who provided the intervention (e.g., physician leader, manager), how the intervention was delivered (mode of delivery - face-to-face vs. videoconference, individual vs. group-based), when and how much the intervention was delivered (number of sessions, schedule, duration, intensity, or dose), any tailoring of the intervention (personalization, titration, or adaptation), and any modifications made during the study (what, why, when, and how). Intervention adherence, both planned and actual, will also be extracted, including assessment methods and strategies to improve fidelity. Additional extracted data will encompass: patient population characteristics, the acute care site characteristics (number of beds, type of service, academic vs. community), rehab site characteristics (number of beds, type of rehab, freestanding vs. non-freestanding rehab hospital or skilled nursing facility, academic vs. community), acute care and rehab provider characteristics (specialty, learners/staff/both), timeframe of handover (before or after admission and the specific timeframe), format of the handover (phone call, videoconference, email), and whether the study aimed to improve discharge summary completion (Yes/No…). Furthermore, we will extract the outcome type (qualitative, quantitative, or both), outcome measures (tools used to assess intervention effectiveness, e.g., % of handovers completed), outcome results, and any balancing measures (unintended consequences of the intervention).

### Data analysis and presentation

The extracted data will be summarized both quantitively with descriptive statistics and qualitatively using content analysis. In the event of missing data, study authors will be contacted for clarification. The synthesis will be conducted in duplicate and involve two synthesists working independently to analyze and code the data manually. Any discrepancies will be reviewed by an additional synthesist. The synthesized data will be available upon reasonable request. Results are expected to be completed by Nov 1^st^, 2025.

## Discussion

This scoping review will be disseminated in multiple avenues, including publication in a peer-reviewed journal and conference presentations. Examples of potential target peer-reviewed journals include PloSOne, Journal of Hospital Medicine, American Journal of Physical Medicine and Rehabilitation, and Journal of Rehabilitation Medicine. Through publication in a peer-reviewed journal, we are aspiring to reach multiple relevant audiences, including healthcare professionals, researchers, professional organizations, policymakers, and other stakeholders who may benefit from the knowledge dissemination. The results from this review will also be presented at various conferences related to hospital medicine, rehabilitation, and patient safety. Some examples of potential target conferences include the American Congress of Rehabilitation Medicine, Canadian Society of Hospital Medicine Conference, and the International Forum on Quality and Safety in Healthcare. These conferences will provide opportunities to engage with multidisciplinary audiences and facilitate discussions on improving physician handover from acute care to rehabilitation settings. In addition, infographics and lay summaries will be created to ensure the findings are accessible to a broader audience through social media.

This scoping review protocol has several methodological strengths. First, it uses a comprehensive scoping review approach through the JBI framework and the PRISMA-ScR guidelines. Second, it involves collaboration with an experienced health sciences librarian which ensures a robust and comprehensive search strategy to minimize the risk of missing relevant studies. Third, all rounds of screening, data extraction, and data synthesis will be conducted independently, with each stage performed in duplicate.

While this review protocol has a number of strengths, this protocol has some limitations. First, the exclusion of full-text studies not written in English may potentially omit insights from non-English speaking healthcare systems. Second, by focusing solely on physician-to-physician handover, this review may overlook contributions from healthcare providers such as nurse practitioners and physician assistants who may practice without physician oversight in some healthcare systems. Third, the exclusion of grey literature and other research publications such as conference abstracts or commentaries may result in omission of insights into real-world challenges and strategies used in clinical practice.

The findings of this review have the potential to benefit multiple stakeholders within the healthcare system. For healthcare professionals, the identification of best practices can help improve handover quality and reduce communication-related errors. Policy makers may use the results to develop policies and training programs aimed at standardizing handover processes. For researchers, this review could provide a foundation for future studies exploring the impact of handover interventions on patient outcomes in rehabilitation. Ultimately, this review could offer important insights in improving the transition of care from acute care to rehabilitation settings to enhance patient safety and improve overall patient outcomes.

## Conclusion

This review is expected to provide valuable insights into the current practice of physician handover strategies in the transition from acute care to rehabilitation. It aims to highlight facilitators and barriers to effective physician-physician handover and suggest innovative approaches that could be implemented in various healthcare settings. By identifying such parameters in the literature, this review has the potential to aid in the development of guidelines such as standardized handover protocols to enhance information transfer from acute care to rehabilitation settings.

## Supporting information

S1 Appendix Search strategy.
(DOCX)

S2 Appendix PRISMA-ScR flow diagram. (DOC)
