# Supplementary material for: Physician-physician handover from acute care to rehabilitation setting: A scoping review protocol
Source: PLoS One. 2025 Dec 12;20(12):e0338916. doi: 10.1371/journal.pone.0338916 (PMC12700418; doi:10.1371/journal.pone.0338916)
Supplement: S1 Appendix — Search strategy. (DOCX) [file pone.0338916.s001.docx]

**S1 Appendix**

Search Strategy Documentation

Scoping Review for Handoffs in Inpatient Rehabilitation Hospitals/Facilities

# Summary of Results

| **Database** | **Interface** | **Date Searched** | **Results** |
| --- | --- | --- | --- |
| MEDLINE(R) ALL | Ovid | February 5, 2025 | 1046 |
| Cochrane Central Register of Controlled Trials | Ovid | February 5, 2025 | 162 |
| Embase Classic + Embase | Ovid | February 5, 2025 | 2815 |
| Emcare | Ovid | February 5, 2025 | 947 |
| PsycInfo | Ovid | February 5, 2025 | 166 |
| CINAHL Ultimate | EBSCO | February 5, 2025 | 1102 |
| Web of Science | Clarivate | February 5, 2025 | 851 |
| TOTAL (prior to de-duplication) |  |  | 7089 |

# Search Strategies

## MEDLINE(R) ALL

**Database:** Ovid MEDLINE(R) ALL <1946 to February 04, 2025>

| # | Query | Results from 5 Feb 2025 |
| --- | --- | --- |
| 1 | Hospitals, Rehabilitation/ | 153 |
| 2 | Rehabilitation Centers/ | 8,838 |
| 3 | Skilled Nursing Facilities/ | 5,519 |
| 4 | ((rehab* or neurorehab* or physiatr* or "physical medicine") adj3 hospital*).tw,kf. | 8,385 |
| 5 | ((rehab* or neurorehab* or physiatr* or "physical medicine") adj3 (inhospital* or "in-hospital*")).tw,kf. | 729 |
| 6 | ((rehab* or neurorehab* or physiatr* or "physical medicine") adj3 (center* or centre*)).tw,kf. | 10,231 |
| 7 | ((rehab* or neurorehab* or physiatr* or "physical medicine") adj3 facilit*).tw,kf. | 5,889 |
| 8 | ((rehab* or neurorehab* or physiatr* or "physical medicine") adj3 (inpatient* or "in-patient*")).tw,kf. | 13,347 |
| 9 | ((rehab* or neurorehab* or physiatr* or "physical medicine") adj3 setting*).tw,kf. | 7,245 |
| 10 | ((rehab* or neurorehab* or physiatr* or "physical medicine") adj3 (ward or wards)).tw,kf. | 1,440 |
| 11 | ((rehab* or neurorehab* or physiatr* or "physical medicine") adj3 (unit or units)).tw,kf. | 4,599 |
| 12 | ((rehab* or neurorehab* or physiatr* or "physical medicine") adj3 department*).tw,kf. | 2,278 |
| 13 | ((rehab* or neurorehab* or physiatr* or "physical medicine") adj3 (admit* or admission*)).tw,kf. | 3,427 |
| 14 | ((rehab* or neurorehab* or physiatr* or "physical medicine") adj3 program*).tw,kf. | 26,712 |
| 15 | ((rehab* or neurorehab* or physiatr* or "physical medicine") adj3 institut*).tw,kf. | 1,397 |
| 16 | ((rehab* or neurorehab* or physiatr* or "physical medicine") adj3 floor?).tw,kf. | 371 |
| 17 | ((rehab* or neurorehab* or physiatr* or "physical medicine") adj3 section*).tw,kf. | 293 |
| 18 | ((rehab* or neurorehab* or physiatr* or "physical medicine") adj3 (bed or beds or bedded)).tw,kf. | 338 |
| 19 | ((rehab* or neurorehab* or physiatr* or "physical medicine") adj3 (stay or stays or stayed or staying)).tw,kf. | 1,457 |
| 20 | ((rehab* or neurorehab* or physiatr* or "physical medicine") adj3 resident?).tw,kf. | 304 |
| 21 | ((rehab* or neurorehab* or physiatr* or "physical medicine") adj3 acute*).tw,kf. | 4,591 |
| 22 | ((rehab* or neurorehab* or physiatr* or "physical medicine") adj3 (post-acute* or postacute*)).tw,kf. | 1,035 |
| 23 | ((rehab* or neurorehab* or physiatr* or "physical medicine") adj3 (sub-acute* or subacute*)).tw,kf. | 747 |
| 24 | ((transfer* to? or transfer* into or transfer* for?) adj3 (rehab* or neurorehab* or physiatr* or "physical medicine")).tw,kf. | 767 |
| 25 | (transfer* adj3 ((to? or into or for?) adj2 (rehab* or neurorehab* or physiatr* or "physical medicine"))).tw,kf. | 726 |
| 26 | ((discharg* to? or discharg* into or discharg* for?) adj3 (rehab* or neurorehab* or physiatr* or "physical medicine")).tw,kf. | 1,540 |
| 27 | (discharg* adj3 ((to? or into or for?) adj2 (rehab* or neurorehab* or physiatr* or "physical medicine"))).tw,kf. | 1,825 |
| 28 | ((transition* to? or transition* into or transition* for?) adj3 (rehab* or neurorehab* or physiatr* or "physical medicine")).tw,kf. | 71 |
| 29 | (transition* adj3 ((to? or into or for?) adj2 (rehab* or neurorehab* or physiatr* or "physical medicine"))).tw,kf. | 164 |
| 30 | skilled nursing.tw,kf. | 4,927 |
| 31 | (extended care adj3 facilit*).tw,kf. | 521 |
| 32 | complex continuing care.tw,kf. | 96 |
| 33 | or/1-32 [Inpatient Rehab Facilities Concept = Concept A] | 79,746 |
| 34 | Patient Handoff/ | 1,737 |
| 35 | Transfer Agreement/ | 259 |
| 36 | handoff*.tw,kf. | 2,042 |
| 37 | (hand? off? or handing off or handed off).tw,kf. | 1,233 |
| 38 | handover*.tw,kf. | 2,482 |
| 39 | (hand? over? or handing over or handed over).tw,kf. | 1,514 |
| 40 | signout*.tw,kf. | 71 |
| 41 | (sign? out? or signing out or signed out).tw,kf. | 793 |
| 42 | signover*.tw,kf. | 1 |
| 43 | (sign? over? or signing over or signed over).tw,kf. | 250 |
| 44 | ("transfer* of?" adj3 care).tw,kf. | 1,016 |
| 45 | (transfer* adj1 care).tw,kf. | 588 |
| 46 | ("transfer* of?" adj3 healthcare).tw,kf. | 53 |
| 47 | (transfer* adj1 healthcare).tw,kf. | 28 |
| 48 | ("transfer* in" adj3 care).tw,kf. | 165 |
| 49 | ("transfer* in" adj3 healthcare).tw,kf. | 15 |
| 50 | (transfer* adj3 (accountabilit* or responsibilit* or authorit* or decision-making)).tw,kf. | 798 |
| 51 | (shift report* or shift-to?-shift or intershift? or inter-shift? or end-of?-shift).tw,kf. | 726 |
| 52 | ((bedside* or bed side*) adj3 report*).tw,kf. | 282 |
| 53 | (physician?-to?-physician? or doctor?-to?-doctor? or hospitalist?-to?-hospitalist? or surgeon?-to?-surgeon? or resident?-to?-resident?).tw,kf. | 397 |
| 54 | (physician?-to?-physiatrist? or doctor?-to?-physiatrist? or hospitalist?-to?-physiatrist? or surgeon?-to?-physiatrist? or resident?-to?-physiatrist? or physician?-to?-doctor? or physician?-to?-hospitalist? or physician?-to?-surgeon? or physician?-to?-resident? or doctor?-to?-physician? or doctor?-to?-hospitalist? or doctor?-to?-surgeon? or doctor?-to?-resident? or hospitalist?-to?-physician? or hospitalist?-to?-doctor? or hospitalist?-to?-surgeon? or hospitalist?-to?-resident? or surgeon?-to?-physician? or surgeon?-to?-doctor? or surgeon?-to?-hospitalist? or surgeon?-to?-resident? or resident?-to?-physician? or resident?-to?-doctor? or resident?-to?-hospitalist? or resident?-to?-surgeon?).tw,kf. | 26 |
| 55 | (provider?-to?-provider? or clinician?-to?-clinician?).tw,kf. | 190 |
| 56 | ((physician?-physician? or doctor?-doctor? or hospitalist?-hospitalist? or surgeon?-surgeon? or resident?-resident?) adj3 (communicat* or document* or transfer* or record* or report* or note? or brief* or consult* or share? or sharing)).tw,kf. | 123 |
| 57 | ((physician?-physiatrist? or doctor?-physiatrist? or hospitalist?-physiatrist? or surgeon?-physiatrist? or resident?-physiatrist? or physician?-doctor? or physician?-hospitalist? or physician?-surgeon? or physician?-resident? or doctor?-physician? or doctor?-hospitalist? or doctor?-surgeon? or doctor?-resident? or hospitalist?-physician? or hospitalist?-doctor? or hospitalist?-surgeon? or hospitalist?-resident? or surgeon?-physician? or surgeon?-doctor? or surgeon?-hospitalist? or surgeon?-resident? or resident?-physician? or resident?-doctor? or resident?-hospitalist? or resident?-surgeon?) adj3 (communicat* or document* or transfer* or record* or report* or note? or brief* or consult* or share? or sharing)).tw,kf. | 283 |
| 58 | ((provider?-provider? or clinician?-clinician?) adj3 (communicat* or document* or transfer* or record* or report* or note? or brief* or consult* or share? or sharing)).tw,kf. | 82 |
| 59 | (transfer* adj2 (agreement* or note? or record* or report* or document* or communicat* or consult*)).tw,kf. | 3,556 |
| 60 | (transfer* adj2 (process* or procedure* or protocol* or checklist* or check list* or mnemonic*)).tw,kf. | 18,733 |
| 61 | (transfer* adj2 (decision* or summar*)).tw,kf. | 645 |
| 62 | (safe* adj3 (transfer* or transition*)).tw,kf. | 2,467 |
| 63 | ("transition? of?" adj2 (care or healthcare)).tw,kf. | 3,113 |
| 64 | ("transition? in" adj2 (care or healthcare)).tw,kf. | 964 |
| 65 | or/34-64 [Handoff Part 1 = Concept B] | 40,376 |
| 66 | Patient Transfer/ | 10,128 |
| 67 | Transitional Care/ | 1,434 |
| 68 | Patient Discharge/ and (Patient Admission/ or Patient Readmission/ or Hospitalization/) | 11,370 |
| 69 | (transfer* adj3 patient*).tw,kf. | 20,044 |
| 70 | (transfer* adj3 (care or healthcare)).tw,kf. | 5,206 |
| 71 | (transfer* adj3 (interfacilit* or inter-facilit* or intrafacilit* or intra-facilit* or interhospital* or inter-hospital* or intrahospital* or intra-hospital* or interward? or inter-ward? or interunit? or inter-unit? or interdepartment* or inter-department*)).tw,kf. | 2,198 |
| 72 | (transfer* adj3 (facilit* or hospital* or ward? or unit? or department* or level? of? care or setting?)).tw,kf. | 21,426 |
| 73 | (transition* adj3 patient*).tw,kf. | 8,024 |
| 74 | (transition* adj3 (care or healthcare)).tw,kf. | 12,910 |
| 75 | (transition* adj3 (interfacilit* or inter-facilit* or intrafacilit* or intra-facilit* or interhospital* or inter-hospital* or intrahospital* or intra-hospital* or interward? or inter-ward? or interunit? or inter-unit? or interdepartment* or inter-department*)).tw,kf. | 30 |
| 76 | (transition* adj3 (facilit* or hospital* or ward? or unit? or department* or level? of? care or setting?)).tw,kf. | 8,485 |
| 77 | ((transfer* to? or transfer* into or transfer* for?) adj3 (rehab* or neurorehab* or physiatr* or "physical medicine" or complex continuing care or skilled nursing or extended care facilit*)).tw,kf. | 837 |
| 78 | (transfer* adj3 ((to? or into or for?) adj2 (rehab* or neurorehab* or physiatr* or "physical medicine" or complex continuing care or skilled nursing or extended care facilit*))).tw,kf. | 805 |
| 79 | ((discharg* to? or discharg* into or discharg* for?) adj3 (rehab* or neurorehab* or physiatr* or "physical medicine" or complex continuing care or skilled nursing or extended care facilit*)).tw,kf. | 2,357 |
| 80 | (discharg* adj3 ((to? or into or for?) adj2 (rehab* or neurorehab* or physiatr* or "physical medicine" or complex continuing care or skilled nursing or extended care facilit*))).tw,kf. | 2,673 |
| 81 | ((transition* to? or transition* into or transition* for?) adj3 (rehab* or neurorehab* or physiatr* or "physical medicine" or complex continuing care or skilled nursing or extended care facilit*)).tw,kf. | 81 |
| 82 | (transition* adj3 ((to? or into or for?) adj2 (rehab* or neurorehab* or physiatr* or "physical medicine" or complex continuing care or skilled nursing or extended care facilit*))).tw,kf. | 214 |
| 83 | or/66-82 [Patient Transfers/Care Transitions = Concept C] | 83,522 |
| 84 | exp Communication/ | 386,036 |
| 85 | Documentation/ | 19,898 |
| 86 | Health Information Exchange/ | 1,165 |
| 87 | communicat*.tw,kf. | 447,522 |
| 88 | document*.tw,kf. | 530,496 |
| 89 | (information* adj4 (exchang* or share? or sharing or transfer* or continuit* or continuum*)).tw,kf. | 38,295 |
| 90 | or/84-89 [Communication/Information Exchange = Concept D] | 1,285,353 |
| 91 | 83 and 90 [Handoff Part 2 = Concept C AND D] | 10,110 |
| 92 | 65 or 91 [Handoff Concept = Concept B OR (C AND D)] | 47,718 |
| 93 | 33 and 92 | 1,046 |

## Cochrane Central Register of Controlled Trials

**Database:** Cochrane Central Register of Controlled Trials <2014 to Present>

| # | Query | Results from 5 Feb 2025 |
| --- | --- | --- |
| 1 | Hospitals, Rehabilitation/ | 10 |
| 2 | Rehabilitation Centers/ | 383 |
| 3 | Skilled Nursing Facilities/ | 138 |
| 4 | ((rehab* or neurorehab* or physiatr* or "physical medicine") adj3 hospital*).tw,kw. | 2,655 |
| 5 | ((rehab* or neurorehab* or physiatr* or "physical medicine") adj3 (inhospital* or "in-hospital*")).tw,kw. | 227 |
| 6 | ((rehab* or neurorehab* or physiatr* or "physical medicine") adj3 (center* or centre*)).tw,kw. | 3,048 |
| 7 | ((rehab* or neurorehab* or physiatr* or "physical medicine") adj3 facilit*).tw,kw. | 808 |
| 8 | ((rehab* or neurorehab* or physiatr* or "physical medicine") adj3 (inpatient* or "in-patient*")).tw,kw. | 3,784 |
| 9 | ((rehab* or neurorehab* or physiatr* or "physical medicine") adj3 setting*).tw,kw. | 1,772 |
| 10 | ((rehab* or neurorehab* or physiatr* or "physical medicine") adj3 (ward or wards)).tw,kw. | 311 |
| 11 | ((rehab* or neurorehab* or physiatr* or "physical medicine") adj3 (unit or units)).tw,kw. | 1,016 |
| 12 | ((rehab* or neurorehab* or physiatr* or "physical medicine") adj3 department*).tw,kw. | 916 |
| 13 | ((rehab* or neurorehab* or physiatr* or "physical medicine") adj3 (admit* or admission*)).tw,kw. | 506 |
| 14 | ((rehab* or neurorehab* or physiatr* or "physical medicine") adj3 program*).tw,kw. | 10,099 |
| 15 | ((rehab* or neurorehab* or physiatr* or "physical medicine") adj3 institut*).tw,kw. | 361 |
| 16 | ((rehab* or neurorehab* or physiatr* or "physical medicine") adj3 floor?).tw,kw. | 198 |
| 17 | ((rehab* or neurorehab* or physiatr* or "physical medicine") adj3 section*).tw,kw. | 36 |
| 18 | ((rehab* or neurorehab* or physiatr* or "physical medicine") adj3 (bed or beds or bedded)).tw,kw. | 46 |
| 19 | ((rehab* or neurorehab* or physiatr* or "physical medicine") adj3 (stay or stays or stayed or staying)).tw,kw. | 334 |
| 20 | ((rehab* or neurorehab* or physiatr* or "physical medicine") adj3 resident?).tw,kw. | 41 |
| 21 | ((rehab* or neurorehab* or physiatr* or "physical medicine") adj3 acute*).tw,kw. | 1,009 |
| 22 | ((rehab* or neurorehab* or physiatr* or "physical medicine") adj3 (post-acute* or postacute*)).tw,kw. | 132 |
| 23 | ((rehab* or neurorehab* or physiatr* or "physical medicine") adj3 (sub-acute* or subacute*)).tw,kw. | 306 |
| 24 | ((transfer* to? or transfer* into or transfer* for?) adj3 (rehab* or neurorehab* or physiatr* or "physical medicine")).tw,kw. | 56 |
| 25 | (transfer* adj3 ((to? or into or for?) adj2 (rehab* or neurorehab* or physiatr* or "physical medicine"))).tw,kw. | 60 |
| 26 | ((discharg* to? or discharg* into or discharg* for?) adj3 (rehab* or neurorehab* or physiatr* or "physical medicine")).tw,kw. | 97 |
| 27 | (discharg* adj3 ((to? or into or for?) adj2 (rehab* or neurorehab* or physiatr* or "physical medicine"))).tw,kw. | 168 |
| 28 | ((transition* to? or transition* into or transition* for?) adj3 (rehab* or neurorehab* or physiatr* or "physical medicine")).tw,kw. | 10 |
| 29 | (transition* adj3 ((to? or into or for?) adj2 (rehab* or neurorehab* or physiatr* or "physical medicine"))).tw,kw. | 26 |
| 30 | skilled nursing.tw,kw. | 337 |
| 31 | (extended care adj3 facilit*).tw,kw. | 34 |
| 32 | complex continuing care.tw,kw. | 5 |
| 33 | or/1-32 [Inpatient Rehab Facilities Concept = Concept A] | 20,658 |
| 34 | Patient Handoff/ | 64 |
| 35 | Transfer Agreement/ | 1 |
| 36 | handoff*.tw,kw. | 125 |
| 37 | (hand? off? or handing off or handed off).tw,kw. | 260 |
| 38 | handover*.tw,kw. | 220 |
| 39 | (hand? over? or handing over or handed over).tw,kw. | 303 |
| 40 | signout*.tw,kw. | 7 |
| 41 | (sign? out? or signing out or signed out).tw,kw. | 58 |
| 42 | signover*.tw,kw. | 0 |
| 43 | (sign? over? or signing over or signed over).tw,kw. | 40 |
| 44 | ("transfer* of?" adj3 care).tw,kw. | 75 |
| 45 | (transfer* adj1 care).tw,kw. | 71 |
| 46 | ("transfer* of?" adj3 healthcare).tw,kw. | 1 |
| 47 | (transfer* adj1 healthcare).tw,kw. | 1 |
| 48 | ("transfer* in" adj3 care).tw,kw. | 13 |
| 49 | ("transfer* in" adj3 healthcare).tw,kw. | 0 |
| 50 | (transfer* adj3 (accountabilit* or responsibilit* or authorit* or decision-making)).tw,kw. | 51 |
| 51 | (shift report* or shift-to?-shift or intershift? or inter-shift? or end-of?-shift).tw,kw. | 41 |
| 52 | ((bedside* or bed side*) adj3 report*).tw,kw. | 17 |
| 53 | (physician?-to?-physician? or doctor?-to?-doctor? or hospitalist?-to?-hospitalist? or surgeon?-to?-surgeon? or resident?-to?-resident?).tw,kw. | 38 |
| 54 | (physician?-to?-physiatrist? or doctor?-to?-physiatrist? or hospitalist?-to?-physiatrist? or surgeon?-to?-physiatrist? or resident?-to?-physiatrist? or physician?-to?-doctor? or physician?-to?-hospitalist? or physician?-to?-surgeon? or physician?-to?-resident? or doctor?-to?-physician? or doctor?-to?-hospitalist? or doctor?-to?-surgeon? or doctor?-to?-resident? or hospitalist?-to?-physician? or hospitalist?-to?-doctor? or hospitalist?-to?-surgeon? or hospitalist?-to?-resident? or surgeon?-to?-physician? or surgeon?-to?-doctor? or surgeon?-to?-hospitalist? or surgeon?-to?-resident? or resident?-to?-physician? or resident?-to?-doctor? or resident?-to?-hospitalist? or resident?-to?-surgeon?).tw,kw. | 2 |
| 55 | (provider?-to?-provider? or clinician?-to?-clinician?).tw,kw. | 16 |
| 56 | ((physician?-physician? or doctor?-doctor? or hospitalist?-hospitalist? or surgeon?-surgeon? or resident?-resident?) adj3 (communicat* or document* or transfer* or record* or report* or note? or brief* or consult* or share? or sharing)).tw,kw. | 12 |
| 57 | ((physician?-physiatrist? or doctor?-physiatrist? or hospitalist?-physiatrist? or surgeon?-physiatrist? or resident?-physiatrist? or physician?-doctor? or physician?-hospitalist? or physician?-surgeon? or physician?-resident? or doctor?-physician? or doctor?-hospitalist? or doctor?-surgeon? or doctor?-resident? or hospitalist?-physician? or hospitalist?-doctor? or hospitalist?-surgeon? or hospitalist?-resident? or surgeon?-physician? or surgeon?-doctor? or surgeon?-hospitalist? or surgeon?-resident? or resident?-physician? or resident?-doctor? or resident?-hospitalist? or resident?-surgeon?) adj3 (communicat* or document* or transfer* or record* or report* or note? or brief* or consult* or share? or sharing)).tw,kw. | 28 |
| 58 | ((provider?-provider? or clinician?-clinician?) adj3 (communicat* or document* or transfer* or record* or report* or note? or brief* or consult* or share? or sharing)).tw,kw. | 8 |
| 59 | (transfer* adj2 (agreement* or note? or record* or report* or document* or communicat* or consult*)).tw,kw. | 224 |
| 60 | (transfer* adj2 (process* or procedure* or protocol* or checklist* or check list* or mnemonic*)).tw,kw. | 373 |
| 61 | (transfer* adj2 (decision* or summar*)).tw,kw. | 89 |
| 62 | (safe* adj3 (transfer* or transition*)).tw,kw. | 289 |
| 63 | ("transition? of?" adj2 (care or healthcare)).tw,kw. | 271 |
| 64 | ("transition? in" adj2 (care or healthcare)).tw,kw. | 80 |
| 65 | or/34-64 [Handoff Part 1 = Concept B] | 2,502 |
| 66 | Patient Transfer/ | 253 |
| 67 | Transitional Care/ | 151 |
| 68 | Patient Discharge/ and (Patient Admission/ or Patient Readmission/ or Hospitalization/) | 776 |
| 69 | (transfer* adj3 patient*).tw,kw. | 2,560 |
| 70 | (transfer* adj3 (care or healthcare)).tw,kw. | 582 |
| 71 | (transfer* adj3 (interfacilit* or inter-facilit* or intrafacilit* or intra-facilit* or interhospital* or inter-hospital* or intrahospital* or intra-hospital* or interward? or inter-ward? or interunit? or inter-unit? or interdepartment* or inter-department*)).tw,kw. | 70 |
| 72 | (transfer* adj3 (facilit* or hospital* or ward? or unit? or department* or level? of? care or setting?)).tw,kw. | 1,286 |
| 73 | (transition* adj3 patient*).tw,kw. | 1,127 |
| 74 | (transition* adj3 (care or healthcare)).tw,kw. | 1,567 |
| 75 | (transition* adj3 (interfacilit* or inter-facilit* or intrafacilit* or intra-facilit* or interhospital* or inter-hospital* or intrahospital* or intra-hospital* or interward? or inter-ward? or interunit? or inter-unit? or interdepartment* or inter-department*)).tw,kw. | 0 |
| 76 | (transition* adj3 (facilit* or hospital* or ward? or unit? or department* or level? of? care or setting?)).tw,kw. | 771 |
| 77 | ((transfer* to? or transfer* into or transfer* for?) adj3 (rehab* or neurorehab* or physiatr* or "physical medicine" or complex continuing care or skilled nursing or extended care facilit*)).tw,kw. | 57 |
| 78 | (transfer* adj3 ((to? or into or for?) adj2 (rehab* or neurorehab* or physiatr* or "physical medicine" or complex continuing care or skilled nursing or extended care facilit*))).tw,kw. | 62 |
| 79 | ((discharg* to? or discharg* into or discharg* for?) adj3 (rehab* or neurorehab* or physiatr* or "physical medicine" or complex continuing care or skilled nursing or extended care facilit*)).tw,kw. | 129 |
| 80 | (discharg* adj3 ((to? or into or for?) adj2 (rehab* or neurorehab* or physiatr* or "physical medicine" or complex continuing care or skilled nursing or extended care facilit*))).tw,kw. | 201 |
| 81 | ((transition* to? or transition* into or transition* for?) adj3 (rehab* or neurorehab* or physiatr* or "physical medicine" or complex continuing care or skilled nursing or extended care facilit*)).tw,kw. | 12 |
| 82 | (transition* adj3 ((to? or into or for?) adj2 (rehab* or neurorehab* or physiatr* or "physical medicine" or complex continuing care or skilled nursing or extended care facilit*))).tw,kw. | 34 |
| 83 | or/66-82 [Patient Transfers/Care Transitions = Concept C] | 7,744 |
| 84 | exp Communication/ | 13,528 |
| 85 | Documentation/ | 273 |
| 86 | Health Information Exchange/ | 17 |
| 87 | communicat*.tw,kw. | 34,623 |
| 88 | document*.tw,kw. | 51,281 |
| 89 | (information* adj4 (exchang* or share? or sharing or transfer* or continuit* or continuum*)).tw,kw. | 1,625 |
| 90 | or/84-89 [Communication/Information Exchange = Concept D] | 94,383 |
| 91 | 83 and 90 [Handoff Part 2 = Concept C AND D] | 1,074 |
| 92 | 65 or 91 [Handoff Concept = Concept B OR (C AND D)] | 3,346 |
| 93 | 33 and 92 | 162 |

## Embase Classic + Embase

**Database:** Embase Classic+Embase <1947 to 2025 February 03>

| # | Query | Results from 5 Feb 2025 |
| --- | --- | --- |
| 1 | rehabilitation center/ | 22,162 |
| 2 | ((rehab* or neurorehab* or physiatr* or "physical medicine") adj3 hospital*).tw,kf. | 13,985 |
| 3 | ((rehab* or neurorehab* or physiatr* or "physical medicine") adj3 (inhospital* or "in-hospital*")).tw,kf. | 1,175 |
| 4 | ((rehab* or neurorehab* or physiatr* or "physical medicine") adj3 (center* or centre*)).tw,kf. | 16,722 |
| 5 | ((rehab* or neurorehab* or physiatr* or "physical medicine") adj3 facilit*).tw,kf. | 10,259 |
| 6 | ((rehab* or neurorehab* or physiatr* or "physical medicine") adj3 (inpatient* or "in-patient*")).tw,kf. | 22,357 |
| 7 | ((rehab* or neurorehab* or physiatr* or "physical medicine") adj3 setting*).tw,kf. | 9,945 |
| 8 | ((rehab* or neurorehab* or physiatr* or "physical medicine") adj3 (ward or wards)).tw,kf. | 2,135 |
| 9 | ((rehab* or neurorehab* or physiatr* or "physical medicine") adj3 (unit or units)).tw,kf. | 8,303 |
| 10 | ((rehab* or neurorehab* or physiatr* or "physical medicine") adj3 department*).tw,kf. | 4,189 |
| 11 | ((rehab* or neurorehab* or physiatr* or "physical medicine") adj3 (admit* or admission*)).tw,kf. | 5,935 |
| 12 | ((rehab* or neurorehab* or physiatr* or "physical medicine") adj3 program*).tw,kf. | 41,798 |
| 13 | ((rehab* or neurorehab* or physiatr* or "physical medicine") adj3 institut*).tw,kf. | 2,461 |
| 14 | ((rehab* or neurorehab* or physiatr* or "physical medicine") adj3 floor?).tw,kf. | 689 |
| 15 | ((rehab* or neurorehab* or physiatr* or "physical medicine") adj3 section*).tw,kf. | 454 |
| 16 | ((rehab* or neurorehab* or physiatr* or "physical medicine") adj3 (bed or beds or bedded)).tw,kf. | 694 |
| 17 | ((rehab* or neurorehab* or physiatr* or "physical medicine") adj3 (stay or stays or stayed or staying)).tw,kf. | 2,527 |
| 18 | ((rehab* or neurorehab* or physiatr* or "physical medicine") adj3 resident?).tw,kf. | 418 |
| 19 | ((rehab* or neurorehab* or physiatr* or "physical medicine") adj3 acute*).tw,kf. | 9,122 |
| 20 | ((rehab* or neurorehab* or physiatr* or "physical medicine") adj3 (post-acute* or postacute*)).tw,kf. | 1,555 |
| 21 | ((rehab* or neurorehab* or physiatr* or "physical medicine") adj3 (sub-acute* or subacute*)).tw,kf. | 1,381 |
| 22 | ((transfer* to? or transfer* into or transfer* for?) adj3 (rehab* or neurorehab* or physiatr* or "physical medicine")).tw,kf. | 1,837 |
| 23 | (transfer* adj3 ((to? or into or for?) adj2 (rehab* or neurorehab* or physiatr* or "physical medicine"))).tw,kf. | 1,535 |
| 24 | ((discharg* to? or discharg* into or discharg* for?) adj3 (rehab* or neurorehab* or physiatr* or "physical medicine")).tw,kf. | 4,233 |
| 25 | (discharg* adj3 ((to? or into or for?) adj2 (rehab* or neurorehab* or physiatr* or "physical medicine"))).tw,kf. | 4,366 |
| 26 | ((transition* to? or transition* into or transition* for?) adj3 (rehab* or neurorehab* or physiatr* or "physical medicine")).tw,kf. | 155 |
| 27 | (transition* adj3 ((to? or into or for?) adj2 (rehab* or neurorehab* or physiatr* or "physical medicine"))).tw,kf. | 286 |
| 28 | skilled nursing.tw,kf. | 8,100 |
| 29 | (extended care adj3 facilit*).tw,kf. | 793 |
| 30 | complex continuing care.tw,kf. | 130 |
| 31 | or/1-30 [Inpatient Rehab Facilities Concept = Concept A] | 126,293 |
| 32 | clinical handover/ | 2,470 |
| 33 | "change of shift report"/ | 56 |
| 34 | handoff*.tw,kf. | 3,051 |
| 35 | (hand? off? or handing off or handed off).tw,kf. | 2,072 |
| 36 | handover*.tw,kf. | 4,350 |
| 37 | (hand? over? or handing over or handed over).tw,kf. | 2,490 |
| 38 | signout*.tw,kf. | 269 |
| 39 | (sign? out? or signing out or signed out).tw,kf. | 1,726 |
| 40 | signover*.tw,kf. | 1 |
| 41 | (sign? over? or signing over or signed over).tw,kf. | 390 |
| 42 | ("transfer* of?" adj3 care).tw,kf. | 1,874 |
| 43 | (transfer* adj1 care).tw,kf. | 1,264 |
| 44 | ("transfer* of?" adj3 healthcare).tw,kf. | 78 |
| 45 | (transfer* adj1 healthcare).tw,kf. | 38 |
| 46 | ("transfer* in" adj3 care).tw,kf. | 259 |
| 47 | ("transfer* in" adj3 healthcare).tw,kf. | 17 |
| 48 | (transfer* adj3 (accountabilit* or responsibilit* or authorit* or decision-making)).tw,kf. | 1,085 |
| 49 | (shift report* or shift-to?-shift or intershift? or inter-shift? or end-of?-shift).tw,kf. | 933 |
| 50 | ((bedside* or bed side*) adj3 report*).tw,kf. | 433 |
| 51 | (physician?-to?-physician? or doctor?-to?-doctor? or hospitalist?-to?-hospitalist? or surgeon?-to?-surgeon? or resident?-to?-resident?).tw,kf. | 550 |
| 52 | (physician?-to?-physiatrist? or doctor?-to?-physiatrist? or hospitalist?-to?-physiatrist? or surgeon?-to?-physiatrist? or resident?-to?-physiatrist? or physician?-to?-doctor? or physician?-to?-hospitalist? or physician?-to?-surgeon? or physician?-to?-resident? or doctor?-to?-physician? or doctor?-to?-hospitalist? or doctor?-to?-surgeon? or doctor?-to?-resident? or hospitalist?-to?-physician? or hospitalist?-to?-doctor? or hospitalist?-to?-surgeon? or hospitalist?-to?-resident? or surgeon?-to?-physician? or surgeon?-to?-doctor? or surgeon?-to?-hospitalist? or surgeon?-to?-resident? or resident?-to?-physician? or resident?-to?-doctor? or resident?-to?-hospitalist? or resident?-to?-surgeon?).tw,kf. | 36 |
| 53 | (provider?-to?-provider? or clinician?-to?-clinician?).tw,kf. | 282 |
| 54 | ((physician?-physician? or doctor?-doctor? or hospitalist?-hospitalist? or surgeon?-surgeon? or resident?-resident?) adj3 (communicat* or document* or transfer* or record* or report* or note? or brief* or consult* or share? or sharing)).tw,kf. | 181 |
| 55 | ((physician?-physiatrist? or doctor?-physiatrist? or hospitalist?-physiatrist? or surgeon?-physiatrist? or resident?-physiatrist? or physician?-doctor? or physician?-hospitalist? or physician?-surgeon? or physician?-resident? or doctor?-physician? or doctor?-hospitalist? or doctor?-surgeon? or doctor?-resident? or hospitalist?-physician? or hospitalist?-doctor? or hospitalist?-surgeon? or hospitalist?-resident? or surgeon?-physician? or surgeon?-doctor? or surgeon?-hospitalist? or surgeon?-resident? or resident?-physician? or resident?-doctor? or resident?-hospitalist? or resident?-surgeon?) adj3 (communicat* or document* or transfer* or record* or report* or note? or brief* or consult* or share? or sharing)).tw,kf. | 417 |
| 56 | ((provider?-provider? or clinician?-clinician?) adj3 (communicat* or document* or transfer* or record* or report* or note? or brief* or consult* or share? or sharing)).tw,kf. | 115 |
| 57 | (transfer* adj2 (agreement* or note? or record* or report* or document* or communicat* or consult*)).tw,kf. | 4,973 |
| 58 | (transfer* adj2 (process* or procedure* or protocol* or checklist* or check list* or mnemonic*)).tw,kf. | 18,978 |
| 59 | (transfer* adj2 (decision* or summar*)).tw,kf. | 1,408 |
| 60 | (safe* adj3 (transfer* or transition*)).tw,kf. | 3,800 |
| 61 | ("transition? of?" adj2 (care or healthcare)).tw,kf. | 5,644 |
| 62 | ("transition? in" adj2 (care or healthcare)).tw,kf. | 1,396 |
| 63 | or/32-62 [Handoff Part 1 = Concept B] | 53,623 |
| 64 | patient transport/ | 36,180 |
| 65 | transitional care/ | 6,416 |
| 66 | hospital discharge/ and (hospital admission/ or hospital readmission/ or hospitalization/) | 83,477 |
| 67 | (transfer* adj3 patient*).tw,kf. | 38,197 |
| 68 | (transfer* adj3 (care or healthcare)).tw,kf. | 9,689 |
| 69 | (transfer* adj3 (interfacilit* or inter-facilit* or intrafacilit* or intra-facilit* or interhospital* or inter-hospital* or intrahospital* or intra-hospital* or interward? or inter-ward? or interunit? or inter-unit? or interdepartment* or inter-department*)).tw,kf. | 3,527 |
| 70 | (transfer* adj3 (facilit* or hospital* or ward? or unit? or department* or level? of? care or setting?)).tw,kf. | 33,137 |
| 71 | (transition* adj3 patient*).tw,kf. | 15,273 |
| 72 | (transition* adj3 (care or healthcare)).tw,kf. | 20,904 |
| 73 | (transition* adj3 (interfacilit* or inter-facilit* or intrafacilit* or intra-facilit* or interhospital* or inter-hospital* or intrahospital* or intra-hospital* or interward? or inter-ward? or interunit? or inter-unit? or interdepartment* or inter-department*)).tw,kf. | 33 |
| 74 | (transition* adj3 (facilit* or hospital* or ward? or unit? or department* or level? of? care or setting?)).tw,kf. | 11,454 |
| 75 | ((transfer* to? or transfer* into or transfer* for?) adj3 (rehab* or neurorehab* or physiatr* or "physical medicine" or complex continuing care or skilled nursing or extended care facilit*)).tw,kf. | 1,996 |
| 76 | (transfer* adj3 ((to? or into or for?) adj2 (rehab* or neurorehab* or physiatr* or "physical medicine" or complex continuing care or skilled nursing or extended care facilit*))).tw,kf. | 1,707 |
| 77 | ((discharg* to? or discharg* into or discharg* for?) adj3 (rehab* or neurorehab* or physiatr* or "physical medicine" or complex continuing care or skilled nursing or extended care facilit*)).tw,kf. | 5,813 |
| 78 | (discharg* adj3 ((to? or into or for?) adj2 (rehab* or neurorehab* or physiatr* or "physical medicine" or complex continuing care or skilled nursing or extended care facilit*))).tw,kf. | 6,035 |
| 79 | ((transition* to? or transition* into or transition* for?) adj3 (rehab* or neurorehab* or physiatr* or "physical medicine" or complex continuing care or skilled nursing or extended care facilit*)).tw,kf. | 180 |
| 80 | (transition* adj3 ((to? or into or for?) adj2 (rehab* or neurorehab* or physiatr* or "physical medicine" or complex continuing care or skilled nursing or extended care facilit*))).tw,kf. | 376 |
| 81 | or/64-80 [Patient Transfers/Care Transitions = Concept C] | 221,950 |
| 82 | exp interpersonal communication/ | 889,486 |
| 83 | documentation/ | 63,257 |
| 84 | medical documentation/ | 31,543 |
| 85 | communicat*.tw,kf. | 586,884 |
| 86 | document*.tw,kf. | 748,584 |
| 87 | (information* adj4 (exchang* or share? or sharing or transfer* or continuit* or continuum*)).tw,kf. | 47,702 |
| 88 | or/82-87 [Communication/Information Exchange = Concept D] | 2,064,066 |
| 89 | 81 and 88 [Handoff Part 2 = Concept C AND D] | 25,546 |
| 90 | 63 or 89 [Handoff Concept = Concept B OR (C AND D)] | 73,910 |
| 91 | 31 and 90 | 2,815 |

## Emcare

**Database:** Ovid Emcare Nursing <1995 to Present>

| # | Query | Results from 5 Feb 2025 |
| --- | --- | --- |
| 1 | rehabilitation center/ | 11,564 |
| 2 | ((rehab* or neurorehab* or physiatr* or "physical medicine") adj3 hospital*).tw,kf. | 5,637 |
| 3 | ((rehab* or neurorehab* or physiatr* or "physical medicine") adj3 (inhospital* or "in-hospital*")).tw,kf. | 473 |
| 4 | ((rehab* or neurorehab* or physiatr* or "physical medicine") adj3 (center* or centre*)).tw,kf. | 6,667 |
| 5 | ((rehab* or neurorehab* or physiatr* or "physical medicine") adj3 facilit*).tw,kf. | 3,814 |
| 6 | ((rehab* or neurorehab* or physiatr* or "physical medicine") adj3 (inpatient* or "in-patient*")).tw,kf. | 9,390 |
| 7 | ((rehab* or neurorehab* or physiatr* or "physical medicine") adj3 setting*).tw,kf. | 5,425 |
| 8 | ((rehab* or neurorehab* or physiatr* or "physical medicine") adj3 (ward or wards)).tw,kf. | 1,079 |
| 9 | ((rehab* or neurorehab* or physiatr* or "physical medicine") adj3 (unit or units)).tw,kf. | 3,422 |
| 10 | ((rehab* or neurorehab* or physiatr* or "physical medicine") adj3 department*).tw,kf. | 1,793 |
| 11 | ((rehab* or neurorehab* or physiatr* or "physical medicine") adj3 (admit* or admission*)).tw,kf. | 2,420 |
| 12 | ((rehab* or neurorehab* or physiatr* or "physical medicine") adj3 program*).tw,kf. | 16,921 |
| 13 | ((rehab* or neurorehab* or physiatr* or "physical medicine") adj3 institut*).tw,kf. | 874 |
| 14 | ((rehab* or neurorehab* or physiatr* or "physical medicine") adj3 floor?).tw,kf. | 163 |
| 15 | ((rehab* or neurorehab* or physiatr* or "physical medicine") adj3 section*).tw,kf. | 223 |
| 16 | ((rehab* or neurorehab* or physiatr* or "physical medicine") adj3 (bed or beds or bedded)).tw,kf. | 220 |
| 17 | ((rehab* or neurorehab* or physiatr* or "physical medicine") adj3 (stay or stays or stayed or staying)).tw,kf. | 1,075 |
| 18 | ((rehab* or neurorehab* or physiatr* or "physical medicine") adj3 resident?).tw,kf. | 224 |
| 19 | ((rehab* or neurorehab* or physiatr* or "physical medicine") adj3 acute*).tw,kf. | 3,272 |
| 20 | ((rehab* or neurorehab* or physiatr* or "physical medicine") adj3 (post-acute* or postacute*)).tw,kf. | 833 |
| 21 | ((rehab* or neurorehab* or physiatr* or "physical medicine") adj3 (sub-acute* or subacute*)).tw,kf. | 564 |
| 22 | ((transfer* to? or transfer* into or transfer* for?) adj3 (rehab* or neurorehab* or physiatr* or "physical medicine")).tw,kf. | 394 |
| 23 | (transfer* adj3 ((to? or into or for?) adj2 (rehab* or neurorehab* or physiatr* or "physical medicine"))).tw,kf. | 381 |
| 24 | ((discharg* to? or discharg* into or discharg* for?) adj3 (rehab* or neurorehab* or physiatr* or "physical medicine")).tw,kf. | 741 |
| 25 | (discharg* adj3 ((to? or into or for?) adj2 (rehab* or neurorehab* or physiatr* or "physical medicine"))).tw,kf. | 960 |
| 26 | ((transition* to? or transition* into or transition* for?) adj3 (rehab* or neurorehab* or physiatr* or "physical medicine")).tw,kf. | 54 |
| 27 | (transition* adj3 ((to? or into or for?) adj2 (rehab* or neurorehab* or physiatr* or "physical medicine"))).tw,kf. | 148 |
| 28 | skilled nursing.tw,kf. | 3,300 |
| 29 | (extended care adj3 facilit*).tw,kf. | 225 |
| 30 | complex continuing care.tw,kf. | 85 |
| 31 | or/1-30 [Inpatient Rehab Facilities Concept = Concept A] | 51,870 |
| 32 | clinical handover/ | 1,237 |
| 33 | "change of shift report"/ | 99 |
| 34 | handoff*.tw,kf. | 1,456 |
| 35 | (hand? off? or handing off or handed off).tw,kf. | 761 |
| 36 | handover*.tw,kf. | 1,794 |
| 37 | (hand? over? or handing over or handed over).tw,kf. | 752 |
| 38 | signout*.tw,kf. | 32 |
| 39 | (sign? out? or signing out or signed out).tw,kf. | 320 |
| 40 | signover*.tw,kf. | 1 |
| 41 | (sign? over? or signing over or signed over).tw,kf. | 87 |
| 42 | ("transfer* of?" adj3 care).tw,kf. | 691 |
| 43 | (transfer* adj1 care).tw,kf. | 415 |
| 44 | ("transfer* of?" adj3 healthcare).tw,kf. | 39 |
| 45 | (transfer* adj1 healthcare).tw,kf. | 18 |
| 46 | ("transfer* in" adj3 care).tw,kf. | 105 |
| 47 | ("transfer* in" adj3 healthcare).tw,kf. | 8 |
| 48 | (transfer* adj3 (accountabilit* or responsibilit* or authorit* or decision-making)).tw,kf. | 563 |
| 49 | (shift report* or shift-to?-shift or intershift? or inter-shift? or end-of?-shift).tw,kf. | 485 |
| 50 | ((bedside* or bed side*) adj3 report*).tw,kf. | 228 |
| 51 | (physician?-to?-physician? or doctor?-to?-doctor? or hospitalist?-to?-hospitalist? or surgeon?-to?-surgeon? or resident?-to?-resident?).tw,kf. | 224 |
| 52 | (physician?-to?-physiatrist? or doctor?-to?-physiatrist? or hospitalist?-to?-physiatrist? or surgeon?-to?-physiatrist? or resident?-to?-physiatrist? or physician?-to?-doctor? or physician?-to?-hospitalist? or physician?-to?-surgeon? or physician?-to?-resident? or doctor?-to?-physician? or doctor?-to?-hospitalist? or doctor?-to?-surgeon? or doctor?-to?-resident? or hospitalist?-to?-physician? or hospitalist?-to?-doctor? or hospitalist?-to?-surgeon? or hospitalist?-to?-resident? or surgeon?-to?-physician? or surgeon?-to?-doctor? or surgeon?-to?-hospitalist? or surgeon?-to?-resident? or resident?-to?-physician? or resident?-to?-doctor? or resident?-to?-hospitalist? or resident?-to?-surgeon?).tw,kf. | 17 |
| 53 | (provider?-to?-provider? or clinician?-to?-clinician?).tw,kf. | 109 |
| 54 | ((physician?-physician? or doctor?-doctor? or hospitalist?-hospitalist? or surgeon?-surgeon? or resident?-resident?) adj3 (communicat* or document* or transfer* or record* or report* or note? or brief* or consult* or share? or sharing)).tw,kf. | 61 |
| 55 | ((physician?-physiatrist? or doctor?-physiatrist? or hospitalist?-physiatrist? or surgeon?-physiatrist? or resident?-physiatrist? or physician?-doctor? or physician?-hospitalist? or physician?-surgeon? or physician?-resident? or doctor?-physician? or doctor?-hospitalist? or doctor?-surgeon? or doctor?-resident? or hospitalist?-physician? or hospitalist?-doctor? or hospitalist?-surgeon? or hospitalist?-resident? or surgeon?-physician? or surgeon?-doctor? or surgeon?-hospitalist? or surgeon?-resident? or resident?-physician? or resident?-doctor? or resident?-hospitalist? or resident?-surgeon?) adj3 (communicat* or document* or transfer* or record* or report* or note? or brief* or consult* or share? or sharing)).tw,kf. | 149 |
| 56 | ((provider?-provider? or clinician?-clinician?) adj3 (communicat* or document* or transfer* or record* or report* or note? or brief* or consult* or share? or sharing)).tw,kf. | 62 |
| 57 | (transfer* adj2 (agreement* or note? or record* or report* or document* or communicat* or consult*)).tw,kf. | 1,081 |
| 58 | (transfer* adj2 (process* or procedure* or protocol* or checklist* or check list* or mnemonic*)).tw,kf. | 2,147 |
| 59 | (transfer* adj2 (decision* or summar*)).tw,kf. | 366 |
| 60 | (safe* adj3 (transfer* or transition*)).tw,kf. | 1,290 |
| 61 | ("transition? of?" adj2 (care or healthcare)).tw,kf. | 2,163 |
| 62 | ("transition? in" adj2 (care or healthcare)).tw,kf. | 721 |
| 63 | or/32-62 [Handoff Part 1 = Concept B] | 14,308 |
| 64 | patient transport/ | 16,534 |
| 65 | transitional care/ | 3,407 |
| 66 | hospital discharge/ and (hospital admission/ or hospital readmission/ or hospitalization/) | 35,779 |
| 67 | (transfer* adj3 patient*).tw,kf. | 9,563 |
| 68 | (transfer* adj3 (care or healthcare)).tw,kf. | 3,343 |
| 69 | (transfer* adj3 (interfacilit* or inter-facilit* or intrafacilit* or intra-facilit* or interhospital* or inter-hospital* or intrahospital* or intra-hospital* or interward? or inter-ward? or interunit? or inter-unit? or interdepartment* or inter-department*)).tw,kf. | 1,398 |
| 70 | (transfer* adj3 (facilit* or hospital* or ward? or unit? or department* or level? of? care or setting?)).tw,kf. | 7,587 |
| 71 | (transition* adj3 patient*).tw,kf. | 3,634 |
| 72 | (transition* adj3 (care or healthcare)).tw,kf. | 9,441 |
| 73 | (transition* adj3 (interfacilit* or inter-facilit* or intrafacilit* or intra-facilit* or interhospital* or inter-hospital* or intrahospital* or intra-hospital* or interward? or inter-ward? or interunit? or inter-unit? or interdepartment* or inter-department*)).tw,kf. | 21 |
| 74 | (transition* adj3 (facilit* or hospital* or ward? or unit? or department* or level? of? care or setting?)).tw,kf. | 4,875 |
| 75 | ((transfer* to? or transfer* into or transfer* for?) adj3 (rehab* or neurorehab* or physiatr* or "physical medicine" or complex continuing care or skilled nursing or extended care facilit*)).tw,kf. | 431 |
| 76 | (transfer* adj3 ((to? or into or for?) adj2 (rehab* or neurorehab* or physiatr* or "physical medicine" or complex continuing care or skilled nursing or extended care facilit*))).tw,kf. | 429 |
| 77 | ((discharg* to? or discharg* into or discharg* for?) adj3 (rehab* or neurorehab* or physiatr* or "physical medicine" or complex continuing care or skilled nursing or extended care facilit*)).tw,kf. | 1,227 |
| 78 | (discharg* adj3 ((to? or into or for?) adj2 (rehab* or neurorehab* or physiatr* or "physical medicine" or complex continuing care or skilled nursing or extended care facilit*))).tw,kf. | 1,465 |
| 79 | ((transition* to? or transition* into or transition* for?) adj3 (rehab* or neurorehab* or physiatr* or "physical medicine" or complex continuing care or skilled nursing or extended care facilit*)).tw,kf. | 62 |
| 80 | (transition* adj3 ((to? or into or for?) adj2 (rehab* or neurorehab* or physiatr* or "physical medicine" or complex continuing care or skilled nursing or extended care facilit*))).tw,kf. | 184 |
| 81 | or/64-80 [Patient Transfers/Care Transitions = Concept C] | 79,078 |
| 82 | exp interpersonal communication/ | 477,567 |
| 83 | documentation/ | 25,359 |
| 84 | medical documentation/ | 15,396 |
| 85 | communicat*.tw,kf. | 218,550 |
| 86 | document*.tw,kf. | 205,298 |
| 87 | (information* adj4 (exchang* or share? or sharing or transfer* or continuit* or continuum*)).tw,kf. | 18,910 |
| 88 | or/82-87 [Communication/Information Exchange = Concept D] | 797,839 |
| 89 | 81 and 88 [Handoff Part 2 = Concept C AND D] | 10,453 |
| 90 | 63 or 89 [Handoff Concept = Concept B OR (C AND D)] | 22,673 |
| 91 | 31 and 90 | 947 |

## PsycInfo

**Database:** APA PsycInfo <1806 to January 2025 Week 4>

| # | Query | Results from 5 Feb 2025 |
| --- | --- | --- |
| 1 | Rehabilitation Centers/ | 805 |
| 2 | ((rehab* or neurorehab* or physiatr* or "physical medicine") adj3 hospital*).tw. | 2,026 |
| 3 | ((rehab* or neurorehab* or physiatr* or "physical medicine") adj3 (inhospital* or "in-hospital*")).tw. | 140 |
| 4 | ((rehab* or neurorehab* or physiatr* or "physical medicine") adj3 (center* or centre*)).tw. | 3,406 |
| 5 | ((rehab* or neurorehab* or physiatr* or "physical medicine") adj3 facilit*).tw. | 1,616 |
| 6 | ((rehab* or neurorehab* or physiatr* or "physical medicine") adj3 (inpatient* or "in-patient*")).tw. | 3,074 |
| 7 | ((rehab* or neurorehab* or physiatr* or "physical medicine") adj3 setting*).tw. | 2,469 |
| 8 | ((rehab* or neurorehab* or physiatr* or "physical medicine") adj3 (ward or wards)).tw. | 352 |
| 9 | ((rehab* or neurorehab* or physiatr* or "physical medicine") adj3 (unit or units)).tw. | 1,354 |
| 10 | ((rehab* or neurorehab* or physiatr* or "physical medicine") adj3 department*).tw. | 413 |
| 11 | ((rehab* or neurorehab* or physiatr* or "physical medicine") adj3 (admit* or admission*)).tw. | 738 |
| 12 | ((rehab* or neurorehab* or physiatr* or "physical medicine") adj3 program*).tw. | 9,813 |
| 13 | ((rehab* or neurorehab* or physiatr* or "physical medicine") adj3 institut*).tw. | 478 |
| 14 | ((rehab* or neurorehab* or physiatr* or "physical medicine") adj3 floor?).tw. | 21 |
| 15 | ((rehab* or neurorehab* or physiatr* or "physical medicine") adj3 section*).tw. | 137 |
| 16 | ((rehab* or neurorehab* or physiatr* or "physical medicine") adj3 (bed or beds or bedded)).tw. | 58 |
| 17 | ((rehab* or neurorehab* or physiatr* or "physical medicine") adj3 (stay or stays or stayed or staying)).tw. | 277 |
| 18 | ((rehab* or neurorehab* or physiatr* or "physical medicine") adj3 resident?).tw. | 93 |
| 19 | ((rehab* or neurorehab* or physiatr* or "physical medicine") adj3 acute*).tw. | 1,169 |
| 20 | ((rehab* or neurorehab* or physiatr* or "physical medicine") adj3 (post-acute* or postacute*)).tw. | 374 |
| 21 | ((rehab* or neurorehab* or physiatr* or "physical medicine") adj3 (sub-acute* or subacute*)).tw. | 180 |
| 22 | ((transfer* to? or transfer* into or transfer* for?) adj3 (rehab* or neurorehab* or physiatr* or "physical medicine")).tw. | 71 |
| 23 | (transfer* adj3 ((to? or into or for?) adj2 (rehab* or neurorehab* or physiatr* or "physical medicine"))).tw. | 75 |
| 24 | ((discharg* to? or discharg* into or discharg* for?) adj3 (rehab* or neurorehab* or physiatr* or "physical medicine")).tw. | 101 |
| 25 | (discharg* adj3 ((to? or into or for?) adj2 (rehab* or neurorehab* or physiatr* or "physical medicine"))).tw. | 185 |
| 26 | ((transition* to? or transition* into or transition* for?) adj3 (rehab* or neurorehab* or physiatr* or "physical medicine")).tw. | 20 |
| 27 | (transition* adj3 ((to? or into or for?) adj2 (rehab* or neurorehab* or physiatr* or "physical medicine"))).tw. | 79 |
| 28 | skilled nursing.tw. | 996 |
| 29 | (extended care adj3 facilit*).tw. | 80 |
| 30 | complex continuing care.tw. | 35 |
| 31 | or/1-30 [Inpatient Rehab Facilities Concept = Concept A] | 22,746 |
| 32 | handoff*.tw. | 356 |
| 33 | (hand? off? or handing off or handed off).tw. | 318 |
| 34 | handover*.tw. | 485 |
| 35 | (hand? over? or handing over or handed over).tw. | 472 |
| 36 | signout*.tw. | 7 |
| 37 | (sign? out? or signing out or signed out).tw. | 70 |
| 38 | signover*.tw. | 0 |
| 39 | (sign? over? or signing over or signed over).tw. | 63 |
| 40 | ("transfer* of?" adj3 care).tw. | 172 |
| 41 | (transfer* adj1 care).tw. | 94 |
| 42 | ("transfer* of?" adj3 healthcare).tw. | 8 |
| 43 | (transfer* adj1 healthcare).tw. | 4 |
| 44 | ("transfer* in" adj3 care).tw. | 26 |
| 45 | ("transfer* in" adj3 healthcare).tw. | 4 |
| 46 | (transfer* adj3 (accountabilit* or responsibilit* or authorit* or decision-making)).tw. | 537 |
| 47 | (shift report* or shift-to?-shift or intershift? or inter-shift? or end-of?-shift).tw. | 159 |
| 48 | ((bedside* or bed side*) adj3 report*).tw. | 39 |
| 49 | (physician?-to?-physician? or doctor?-to?-doctor? or hospitalist?-to?-hospitalist? or surgeon?-to?-surgeon? or resident?-to?-resident?).tw. | 93 |
| 50 | (physician?-to?-physiatrist? or doctor?-to?-physiatrist? or hospitalist?-to?-physiatrist? or surgeon?-to?-physiatrist? or resident?-to?-physiatrist? or physician?-to?-doctor? or physician?-to?-hospitalist? or physician?-to?-surgeon? or physician?-to?-resident? or doctor?-to?-physician? or doctor?-to?-hospitalist? or doctor?-to?-surgeon? or doctor?-to?-resident? or hospitalist?-to?-physician? or hospitalist?-to?-doctor? or hospitalist?-to?-surgeon? or hospitalist?-to?-resident? or surgeon?-to?-physician? or surgeon?-to?-doctor? or surgeon?-to?-hospitalist? or surgeon?-to?-resident? or resident?-to?-physician? or resident?-to?-doctor? or resident?-to?-hospitalist? or resident?-to?-surgeon?).tw. | 3 |
| 51 | (provider?-to?-provider? or clinician?-to?-clinician?).tw. | 47 |
| 52 | ((physician?-physician? or doctor?-doctor? or hospitalist?-hospitalist? or surgeon?-surgeon? or resident?-resident?) adj3 (communicat* or document* or transfer* or record* or report* or note? or brief* or consult* or share? or sharing)).tw. | 33 |
| 53 | ((physician?-physiatrist? or doctor?-physiatrist? or hospitalist?-physiatrist? or surgeon?-physiatrist? or resident?-physiatrist? or physician?-doctor? or physician?-hospitalist? or physician?-surgeon? or physician?-resident? or doctor?-physician? or doctor?-hospitalist? or doctor?-surgeon? or doctor?-resident? or hospitalist?-physician? or hospitalist?-doctor? or hospitalist?-surgeon? or hospitalist?-resident? or surgeon?-physician? or surgeon?-doctor? or surgeon?-hospitalist? or surgeon?-resident? or resident?-physician? or resident?-doctor? or resident?-hospitalist? or resident?-surgeon?) adj3 (communicat* or document* or transfer* or record* or report* or note? or brief* or consult* or share? or sharing)).tw. | 65 |
| 54 | ((provider?-provider? or clinician?-clinician?) adj3 (communicat* or document* or transfer* or record* or report* or note? or brief* or consult* or share? or sharing)).tw. | 41 |
| 55 | (transfer* adj2 (agreement* or note? or record* or report* or document* or communicat* or consult*)).tw. | 644 |
| 56 | (transfer* adj2 (process* or procedure* or protocol* or checklist* or check list* or mnemonic*)).tw. | 2,061 |
| 57 | (transfer* adj2 (decision* or summar*)).tw. | 252 |
| 58 | (safe* adj3 (transfer* or transition*)).tw. | 389 |
| 59 | ("transition? of?" adj2 (care or healthcare)).tw. | 479 |
| 60 | ("transition? in" adj2 (care or healthcare)).tw. | 220 |
| 61 | or/32-60 [Handoff Part 1 = Concept B] | 6,567 |
| 62 | Client Transfer/ | 314 |
| 63 | exp Facility Discharge/ and (exp Facility Admission/ or Hospitalization/) | 1,031 |
| 64 | (transfer* adj3 patient*).tw. | 2,405 |
| 65 | (transfer* adj3 (care or healthcare)).tw. | 803 |
| 66 | (transfer* adj3 (interfacilit* or inter-facilit* or intrafacilit* or intra-facilit* or interhospital* or inter-hospital* or intrahospital* or intra-hospital* or interward? or inter-ward? or interunit? or inter-unit? or interdepartment* or inter-department*)).tw. | 74 |
| 67 | (transfer* adj3 (facilit* or hospital* or ward? or unit? or department* or level? of? care or setting?)).tw. | 2,762 |
| 68 | (transition* adj3 patient*).tw. | 1,208 |
| 69 | (transition* adj3 (care or healthcare)).tw. | 3,533 |
| 70 | (transition* adj3 (interfacilit* or inter-facilit* or intrafacilit* or intra-facilit* or interhospital* or inter-hospital* or intrahospital* or intra-hospital* or interward? or inter-ward? or interunit? or inter-unit? or interdepartment* or inter-department*)).tw. | 9 |
| 71 | (transition* adj3 (facilit* or hospital* or ward? or unit? or department* or level? of? care or setting?)).tw. | 3,284 |
| 72 | ((transfer* to? or transfer* into or transfer* for?) adj3 (rehab* or neurorehab* or physiatr* or "physical medicine" or complex continuing care or skilled nursing or extended care facilit*)).tw. | 76 |
| 73 | (transfer* adj3 ((to? or into or for?) adj2 (rehab* or neurorehab* or physiatr* or "physical medicine" or complex continuing care or skilled nursing or extended care facilit*))).tw. | 81 |
| 74 | ((discharg* to? or discharg* into or discharg* for?) adj3 (rehab* or neurorehab* or physiatr* or "physical medicine" or complex continuing care or skilled nursing or extended care facilit*)).tw. | 148 |
| 75 | (discharg* adj3 ((to? or into or for?) adj2 (rehab* or neurorehab* or physiatr* or "physical medicine" or complex continuing care or skilled nursing or extended care facilit*))).tw. | 238 |
| 76 | ((transition* to? or transition* into or transition* for?) adj3 (rehab* or neurorehab* or physiatr* or "physical medicine" or complex continuing care or skilled nursing or extended care facilit*)).tw. | 22 |
| 77 | (transition* adj3 ((to? or into or for?) adj2 (rehab* or neurorehab* or physiatr* or "physical medicine" or complex continuing care or skilled nursing or extended care facilit*))).tw. | 90 |
| 78 | or/62-77 [Patient Transfers/Care Transitions = Concept C] | 13,675 |
| 79 | exp Communication/ | 418,925 |
| 80 | communicat*.tw. | 277,034 |
| 81 | document*.tw. | 125,852 |
| 82 | (information* adj4 (exchang* or share? or sharing or transfer* or continuit* or continuum*)).tw. | 17,163 |
| 83 | or/79-82 [Communication/Information Exchange = Concept D] | 701,640 |
| 84 | 78 and 83 [Handoff Part 2 = Concept C AND D] | 2,353 |
| 85 | 61 or 84 [Handoff Concept = Concept B OR (C AND D)] | 8,405 |
| 86 | 31 and 85 | 166 |

## CINAHL Ultimate

**Database:** CINAHL Ultimate (EBSCOhost)

| # | Query | Limiters/Expanders | Results |
| --- | --- | --- | --- |
| S1 | (MH "Rehabilitation Centers+") | Expanders - Apply equivalent subjects Search modes - Proximity | 10,085 |
| S2 | (MH "Skilled Nursing Facilities") | Expanders - Apply equivalent subjects Search modes - Proximity | 4,960 |
| S3 | TI ((rehab* OR neurorehab* OR physiatr* OR "physical medicine") N2 hospital*) OR AB ((rehab* OR neurorehab* OR physiatr* OR "physical medicine") N2 hospital*) | Expanders - Apply equivalent subjects Search modes - Proximity | 4,754 |
| S4 | TI ((rehab* OR neurorehab* OR physiatr* OR "physical medicine") N2 (inhospital* OR in-hospital*)) OR AB ((rehab* OR neurorehab* OR physiatr* OR "physical medicine") N2 (inhospital* OR in-hospital*)) | Expanders - Apply equivalent subjects Search modes - Proximity | 350 |
| S5 | TI ((rehab* OR neurorehab* OR physiatr* OR "physical medicine") N2 (center* OR centre*)) OR AB ((rehab* OR neurorehab* OR physiatr* OR "physical medicine") N2 (center* OR centre*)) | Expanders - Apply equivalent subjects Search modes - Proximity | 5,598 |
| S6 | TI ((rehab* OR neurorehab* OR physiatr* OR "physical medicine") N2 facilit*) OR AB ((rehab* OR neurorehab* OR physiatr* OR "physical medicine") N2 facilit*) | Expanders - Apply equivalent subjects Search modes - Proximity | 3,319 |
| S7 | TI ((rehab* OR neurorehab* OR physiatr* OR "physical medicine") N2 (inpatient* OR "in-patient*")) OR AB ((rehab* OR neurorehab* OR physiatr* OR "physical medicine") N2 (inpatient* OR "in-patient*")) | Expanders - Apply equivalent subjects Search modes - Proximity | 8,473 |
| S8 | TI ((rehab* OR neurorehab* OR physiatr* OR "physical medicine") N2 setting*) OR AB ((rehab* OR neurorehab* OR physiatr* OR "physical medicine") N2 setting*) | Expanders - Apply equivalent subjects Search modes - Proximity | 5,139 |
| S9 | TI ((rehab* OR neurorehab* OR physiatr* OR "physical medicine") N2 (ward OR wards)) OR AB ((rehab* OR neurorehab* OR physiatr* OR "physical medicine") N2 (ward OR wards)) | Expanders - Apply equivalent subjects Search modes - Proximity | 842 |
| S10 | TI ((rehab* OR neurorehab* OR physiatr* OR "physical medicine") N2 (unit OR units)) OR AB ((rehab* OR neurorehab* OR physiatr* OR "physical medicine") N2 (unit OR units)) | Expanders - Apply equivalent subjects Search modes - Proximity | 3,059 |
| S11 | TI ((rehab* OR neurorehab* OR physiatr* OR "physical medicine") N2 department*) OR AB ((rehab* OR neurorehab* OR physiatr* OR "physical medicine") N2 department*) | Expanders - Apply equivalent subjects Search modes - Proximity | 1,079 |
| S12 | TI ((rehab* OR neurorehab* OR physiatr* OR "physical medicine") N2 (admit* OR admission*)) OR AB ((rehab* OR neurorehab* OR physiatr* OR "physical medicine") N2 (admit* OR admission*)) | Expanders - Apply equivalent subjects Search modes - Proximity | 2,072 |
| S13 | TI ((rehab* OR neurorehab* OR physiatr* OR "physical medicine") N2 program*) OR AB ((rehab* OR neurorehab* OR physiatr* OR "physical medicine") N2 program*) | Expanders - Apply equivalent subjects Search modes - Proximity | 14,435 |
| S14 | TI ((rehab* OR neurorehab* OR physiatr* OR "physical medicine") N2 institut*) OR AB ((rehab* OR neurorehab* OR physiatr* OR "physical medicine") N2 institut*) | Expanders - Apply equivalent subjects Search modes - Proximity | 741 |
| S15 | TI ((rehab* OR neurorehab* OR physiatr* OR "physical medicine") N2 floor#) OR AB ((rehab* OR neurorehab* OR physiatr* OR "physical medicine") N2 floor#) | Expanders - Apply equivalent subjects Search modes - Proximity | 156 |
| S16 | TI ((rehab* OR neurorehab* OR physiatr* OR "physical medicine") N2 section*) OR AB ((rehab* OR neurorehab* OR physiatr* OR "physical medicine") N2 section*) | Expanders - Apply equivalent subjects Search modes - Proximity | 292 |
| S17 | TI ((rehab* OR neurorehab* OR physiatr* OR "physical medicine") N2 (bed OR beds OR bedded)) OR AB ((rehab* OR neurorehab* OR physiatr* OR "physical medicine") N2 (bed OR beds OR bedded)) | Expanders - Apply equivalent subjects Search modes - Proximity | 131 |
| S18 | TI ((rehab* OR neurorehab* OR physiatr* OR "physical medicine") N2 (stay OR stays OR stayed OR staying)) OR AB ((rehab* OR neurorehab* OR physiatr* OR "physical medicine") N2 (stay OR stays OR stayed OR staying)) | Expanders - Apply equivalent subjects Search modes - Proximity | 875 |
| S19 | TI ((rehab* OR neurorehab* OR physiatr* OR "physical medicine") N2 resident#) OR AB ((rehab* OR neurorehab* OR physiatr* OR "physical medicine") N2 resident#) | Expanders - Apply equivalent subjects Search modes - Proximity | 220 |
| S20 | TI ((rehab* OR neurorehab* OR physiatr* OR "physical medicine") N2 acute*) OR AB ((rehab* OR neurorehab* OR physiatr* OR "physical medicine") N2 acute*) | Expanders - Apply equivalent subjects Search modes - Proximity | 3,277 |
| S21 | TI ((rehab* OR neurorehab* OR physiatr* OR "physical medicine") N2 (post-acute* OR postacute*)) OR AB ((rehab* OR neurorehab* OR physiatr* OR "physical medicine") N2 (post-acute* OR postacute*)) | Expanders - Apply equivalent subjects Search modes - Proximity | 758 |
| S22 | TI ((rehab* OR neurorehab* OR physiatr* OR "physical medicine") N2 (sub-acute* OR subacute*)) OR AB ((rehab* OR neurorehab* OR physiatr* OR "physical medicine") N2 (sub-acute* OR subacute*)) | Expanders - Apply equivalent subjects Search modes - Proximity | 505 |
| S23 | TI (("transfer* to" OR "transfer* into") N2 (rehab* OR neurorehab* OR physiatr* OR "physical medicine")) OR AB (("transfer* to" OR "transfer* into") N2 (rehab* OR neurorehab* OR physiatr* OR "physical medicine")) | Expanders - Apply equivalent subjects Search modes - Proximity | 299 |
| S24 | TI ("transfer* for" W2 (rehab* OR neurorehab* OR physiatr* OR "physical medicine")) OR AB ("transfer* for" W2 (rehab* OR neurorehab* OR physiatr* OR "physical medicine")) | Expanders - Apply equivalent subjects Search modes - Proximity | 399 |
| S25 | TI (transfer* N2 ((to OR into) N1 (rehab* OR neurorehab* OR physiatr* OR "physical medicine"))) OR AB (transfer* N2 ((to OR into) N1 (rehab* OR neurorehab* OR physiatr* OR "physical medicine"))) | Expanders - Apply equivalent subjects Search modes - Proximity | 261 |
| S26 | TI (transfer* W2 (for W1 (rehab* OR neurorehab* OR physiatr* OR "physical medicine"))) OR AB (transfer* W2 (for W1 (rehab* OR neurorehab* OR physiatr* OR "physical medicine"))) | Expanders - Apply equivalent subjects Search modes - Proximity | 470 |
| S27 | TI (("discharg* to" OR "discharg* into") N2 (rehab* OR neurorehab* OR physiatr* OR "physical medicine")) OR AB (("discharg* to" OR "discharg* into") N2 (rehab* OR neurorehab* OR physiatr* OR "physical medicine")) | Expanders - Apply equivalent subjects Search modes - Proximity | 628 |
| S28 | TI ("discharg* for" W2 (rehab* OR neurorehab* OR physiatr* OR "physical medicine")) OR AB ("discharg* for" W2 (rehab* OR neurorehab* OR physiatr* OR "physical medicine")) | Expanders - Apply equivalent subjects Search modes - Proximity | 2,154 |
| S29 | TI (discharg* N2 ((to OR into) N1 (rehab* OR neurorehab* OR physiatr* OR "physical medicine"))) OR AB (discharg* N2 ((to OR into) N1 (rehab* OR neurorehab* OR physiatr* OR "physical medicine"))) | Expanders - Apply equivalent subjects Search modes - Proximity | 703 |
| S30 | TI (discharg* W2 (for W1 (rehab* OR neurorehab* OR physiatr* OR "physical medicine"))) OR AB (discharg* W2 (for W1 (rehab* OR neurorehab* OR physiatr* OR "physical medicine"))) | Expanders - Apply equivalent subjects Search modes - Proximity | 2,522 |
| S31 | TI ((“transition* to” OR “transition* into”) N2 (rehab* OR neurorehab* OR physiatr* OR "physical medicine")) OR AB ((“transition* to” OR “transition* into”) N2 (rehab* OR neurorehab* OR physiatr* OR "physical medicine")) | Expanders - Apply equivalent subjects Search modes - Proximity | 51 |
| S32 | TI ((“transition* for”) W2 (rehab* OR neurorehab* OR physiatr* OR "physical medicine")) OR AB ((“transition* for”) W2 (rehab* OR neurorehab* OR physiatr* OR "physical medicine")) | Expanders - Apply equivalent subjects Search modes - Proximity | 217 |
| S33 | TI (transition* N2 ((to OR into) N1 (rehab* OR neurorehab* OR physiatr* OR "physical medicine"))) OR AB (transition* N2 ((to OR into) N1 (rehab* OR neurorehab* OR physiatr* OR "physical medicine"))) | Expanders - Apply equivalent subjects Search modes - Proximity | 104 |
| S34 | TI (transition* W2 (for W1 (rehab* OR neurorehab* OR physiatr* OR "physical medicine"))) OR AB (transition* W2 (for W1 (rehab* OR neurorehab* OR physiatr* OR "physical medicine"))) | Expanders - Apply equivalent subjects Search modes - Proximity | 271 |
| S35 | TI ("skilled nursing") OR AB ("skilled nursing") | Expanders - Apply equivalent subjects Search modes - Proximity | 3,526 |
| S36 | TI ("extended care" N2 facilit*) OR AB ("extended care" N2 facilit*) | Expanders - Apply equivalent subjects Search modes - Proximity | 271 |
| S37 | TI ("complex continuing care") OR AB ("complex continuing care") | Expanders - Apply equivalent subjects Search modes - Proximity | 89 |
| S38 | S1 OR S2 OR S3 OR S4 OR S5 OR S6 OR S7 OR S8 OR S9 OR S10 OR S11 OR S12 OR S13 OR S14 OR S15 OR S16 OR S17 OR S18 OR S19 OR S20 OR S21 OR S22 OR S23 OR S24 OR S25 OR S26 OR S27 OR S28 OR S29 OR S30 OR S31 OR S32 OR S33 OR S34 OR S35 OR S36 OR S37 | Expanders - Apply equivalent subjects Search modes - Proximity | 50,359 |
| S39 | (MH "Hand Off (Patient Safety)+") | Expanders - Apply equivalent subjects Search modes - Proximity | 3,037 |
| S40 | (MH "Shift Reports") | Expanders - Apply equivalent subjects Search modes - Proximity | 934 |
| S41 | TI (handoff*) OR AB (handoff*) | Expanders - Apply equivalent subjects Search modes - Proximity | 1,469 |
| S42 | TI ("hand# off#" OR "handing off" OR "handed off") OR AB ("hand# off#" OR "handing off" OR "handed off") | Expanders - Apply equivalent subjects Search modes - Proximity | 698 |
| S43 | TI (handover*) OR AB (handover*) | Expanders - Apply equivalent subjects Search modes - Proximity | 1,648 |
| S44 | TI ("hand# over#" OR "handing over" OR "handed over") OR AB ("hand# over#" OR "handing over" OR "handed over") | Expanders - Apply equivalent subjects Search modes - Proximity | 531 |
| S45 | TI (signout*) OR AB (signout*) | Expanders - Apply equivalent subjects Search modes - Proximity | 28 |
| S46 | TI ("sign# out#" OR "signing out" OR "signed out") OR AB ("sign# out#" OR "signing out" OR "signed out") | Expanders - Apply equivalent subjects Search modes - Proximity | 298 |
| S47 | TI (signover*) OR AB (signover*) | Expanders - Apply equivalent subjects Search modes - Proximity | 0 |
| S48 | TI ("sign# over#" OR "signing over" OR "signed over") OR AB ("sign# over#" OR "signing over" OR "signed over") | Expanders - Apply equivalent subjects Search modes - Proximity | 62 |
| S49 | TI ("transfer* of" W2 care) OR AB ("transfer* of" W2 care) | Expanders - Apply equivalent subjects Search modes - Proximity | 2,848 |
| S50 | TI (transfer* N0 care) OR AB (transfer* N0 care) | Expanders - Apply equivalent subjects Search modes - Proximity | 364 |
| S51 | TI ("transfer* of" W2 healthcare) OR AB ("transfer* of" W2 healthcare) | Expanders - Apply equivalent subjects Search modes - Proximity | 340 |
| S52 | TI (transfer* N0 healthcare) OR AB (transfer* N0 healthcare) | Expanders - Apply equivalent subjects Search modes - Proximity | 29 |
| S53 | TI ("transfer* in" N2 care) OR AB ("transfer* in" N2 care) | Expanders - Apply equivalent subjects Search modes - Proximity | 69 |
| S54 | TI ("transfer* in" N2 healthcare) OR AB ("transfer* in" N2 healthcare) | Expanders - Apply equivalent subjects Search modes - Proximity | 9 |
| S55 | TI (transfer* N2 (accountabilit* OR responsibilit* OR authorit* OR decision-making)) OR AB (transfer* N2 (accountabilit* OR responsibilit* OR authorit* OR decision-making)) | Expanders - Apply equivalent subjects Search modes - Proximity | 437 |
| S56 | TI ("shift report*" OR shift-to-shift OR intershift# OR inter-shift# OR end-of-shift) OR AB ("shift report*" OR shift-to-shift OR intershift# OR inter-shift# OR end-of-shift) | Expanders - Apply equivalent subjects Search modes - Proximity | 1,307 |
| S57 | TI ((bedside* OR "bed side*") N2 report*) OR AB ((bedside* OR "bed side*") N2 report*) | Expanders - Apply equivalent subjects Search modes - Proximity | 232 |
| S58 | TI (physician#-to-physician# OR doctor#-to-doctor# OR hospitalist#-to-hospitalist# OR surgeon#-to-surgeon# OR resident#-to-resident#) OR AB (physician#-to-physician# OR doctor#-to-doctor# OR hospitalist#-to-hospitalist# OR surgeon#-to-surgeon# OR resident#-to-resident#) | Expanders - Apply equivalent subjects Search modes - Proximity | 199 |
| S59 | TI (physician#-to-physiatrist# OR doctor#-to-physiatrist# OR hospitalist#-to-physiatrist# OR surgeon#-to-physiatrist# OR resident#-to-physiatrist# OR physician#-to-doctor# OR physician#-to-hospitalist# OR physician#-to-surgeon# OR physician#-to-resident# OR doctor#-to-physician# OR doctor#-to-hospitalist# OR doctor#-to-surgeon# OR doctor#-to-resident# OR hospitalist#-to-physician# OR hospitalist#-to-doctor# OR hospitalist#-to-surgeon# OR hospitalist#-to-resident# OR surgeon#-to-physician# OR surgeon#-to-doctor# OR surgeon#-to-hospitalist# OR surgeon#-to-resident# OR resident#-to-physician# OR resident#-to-doctor# OR resident#-to-hospitalist# OR resident#-to-surgeon#) OR AB (physician#-to-physiatrist# OR doctor#-to-physiatrist# OR hospitalist#-to-physiatrist# OR surgeon#-to-physiatrist# OR resident#-to-physiatrist# OR physician#-to-doctor# OR physician#-to-hospitalist# OR physician#-to-surgeon# OR physician#-to-resident# OR doctor#-to-physician# OR doctor#-to-hospitalist# OR doctor#-to-surgeon# OR doctor#-to-resident# OR hospitalist#-to-physician# OR hospitalist#-to-doctor# OR hospitalist#-to-surgeon# OR hospitalist#-to-resident# OR surgeon#-to-physician# OR surgeon#-to-doctor# OR surgeon#-to-hospitalist# OR surgeon#-to-resident# OR resident#-to-physician# OR resident#-to-doctor# OR resident#-to-hospitalist# OR resident#-to-surgeon#) | Expanders - Apply equivalent subjects Search modes - Proximity | 13 |
| S60 | TI (provider#-to-provider# OR clinician#-to-clinician#) OR AB (provider#-to-provider# OR clinician#-to-clinician#) | Expanders - Apply equivalent subjects Search modes - Proximity | 110 |
| S61 | TI ((physician#-physician# OR doctor#-doctor# OR hospitalist#-hospitalist# OR surgeon#-surgeon# OR resident#-resident#) N2 (communicat* OR document* OR transfer* OR record* OR report* OR note# OR brief* OR consult* OR share# OR sharing)) OR AB ((physician#-physician# OR doctor#-doctor# OR hospitalist#-hospitalist# OR surgeon#-surgeon# OR resident#-resident#) N2 (communicat* OR document* OR transfer* OR record* OR report* OR note# OR brief* OR consult* OR share# OR sharing)) | Expanders - Apply equivalent subjects Search modes - Proximity | 45 |
| S62 | TI ((physician#-physiatrist# OR doctor#-physiatrist# OR hospitalist#-physiatrist# OR surgeon#-physiatrist# OR resident#-physiatrist# OR physician#-doctor# OR physician#-hospitalist# OR physician#-surgeon# OR physician#-resident# OR doctor#-physician# OR doctor#-hospitalist# OR doctor#-surgeon# OR doctor#-resident# OR hospitalist#-physician# OR hospitalist#-doctor# OR hospitalist#-surgeon# OR hospitalist#-resident# OR surgeon#-physician# OR surgeon#-doctor# OR surgeon#-hospitalist# OR surgeon#-resident# OR resident#-physician# OR resident#-doctor# OR resident#-hospitalist# OR resident#-surgeon#) N2 (communicat* OR document* OR transfer* OR record* OR report* OR note# OR brief* OR consult* OR share# OR sharing)) OR AB ((physician#-physiatrist# OR doctor#-physiatrist# OR hospitalist#-physiatrist# OR surgeon#-physiatrist# OR resident#-physiatrist# OR physician#-doctor# OR physician#-hospitalist# OR physician#-surgeon# OR physician#-resident# OR doctor#-physician# OR doctor#-hospitalist# OR doctor#-surgeon# OR doctor#-resident# OR hospitalist#-physician# OR hospitalist#-doctor# OR hospitalist#-surgeon# OR hospitalist#-resident# OR surgeon#-physician# OR surgeon#-doctor# OR surgeon#-hospitalist# OR surgeon#-resident# OR resident#-physician# OR resident#-doctor# OR resident#-hospitalist# OR resident#-surgeon#) N2 (communicat* OR document* OR transfer* OR record* OR report* OR note# OR brief* OR consult* OR share# OR sharing)) | Expanders - Apply equivalent subjects Search modes - Proximity | 115 |
| S63 | TI ((provider#-provider# OR clinician#-clinician#) N2 (communicat* OR document* OR transfer* OR record* OR report* OR note# OR brief* OR consult* OR share# OR sharing)) OR AB ((provider#-provider# OR clinician#-clinician#) N2 (communicat* OR document* OR transfer* OR record* OR report* OR note# OR brief* OR consult* OR share# OR sharing)) | Expanders - Apply equivalent subjects Search modes - Proximity | 52 |
| S64 | TI (transfer* N1 (agreement* OR note# OR record* OR report* OR document* OR communicat* OR consult*)) OR AB (transfer* N1 (agreement* OR note# OR record* OR report* OR document* OR communicat* OR consult*)) | Expanders - Apply equivalent subjects Search modes - Proximity | 698 |
| S65 | TI (transfer* N1 (process* OR procedure* OR protocol* OR checklist* OR "check list*" OR mnemonic*)) OR AB (transfer* N1 (process* OR procedure* OR protocol* OR checklist* OR "check list*" OR mnemonic*)) | Expanders - Apply equivalent subjects Search modes - Proximity | 1,074 |
| S66 | TI (transfer* N1 (decision* OR summar*)) OR AB (transfer* N1 (decision* OR summar*)) | Expanders - Apply equivalent subjects Search modes - Proximity | 313 |
| S67 | TI (safe* N2 (transfer* OR transition*)) OR AB (safe* N2 (transfer* OR transition*)) | Expanders - Apply equivalent subjects Search modes - Proximity | 1,089 |
| S68 | TI ("transition# of" W1 (care OR healthcare)) OR AB ("transition# of" W1 (care OR healthcare)) | Expanders - Apply equivalent subjects Search modes - Proximity | 3,802 |
| S69 | TI ("transition# in" N1 (care OR healthcare)) OR AB ("transition# in" N1 (care OR healthcare)) | Expanders - Apply equivalent subjects Search modes - Proximity | 592 |
| S70 | S39 OR S40 OR S41 OR S42 OR S43 OR S44 OR S45 OR S46 OR S47 OR S48 OR S49 OR S50 OR S51 OR S52 OR S53 OR S54 OR S55 OR S56 OR S57 OR S58 OR S59 OR S60 OR S61 OR S62 OR S63 OR S64 OR S65 OR S66 OR S67 OR S68 OR S69 | Expanders - Apply equivalent subjects Search modes - Proximity | 17,235 |
| S71 | (MH "Transfer, Discharge") | Expanders - Apply equivalent subjects Search modes - Proximity | 6,856 |
| S72 | (MH "Transfer, Intrahospital") | Expanders - Apply equivalent subjects Search modes - Proximity | 1,372 |
| S73 | (MH "Transitional Care") | Expanders - Apply equivalent subjects Search modes - Proximity | 4,597 |
| S74 | (MH "Patient Discharge") AND ((MH "Patient Admission") OR (MH "Readmission") OR (MH "Hospitalization")) | Expanders - Apply equivalent subjects Search modes - Proximity | 7,614 |
| S75 | TI (transfer* N2 patient*) OR AB (transfer* N2 patient*) | Expanders - Apply equivalent subjects Search modes - Proximity | 6,447 |
| S76 | TI (transfer* N2 (care OR healthcare)) OR AB (transfer* N2 (care OR healthcare)) | Expanders - Apply equivalent subjects Search modes - Proximity | 2,721 |
| S77 | TI (transfer* N2 (interfacilit* OR inter-facilit* OR intrafacilit* OR intra-facilit* OR interhospital* OR inter-hospital* OR intrahospital* OR intra-hospital* OR interward# OR inter-ward# OR interunit# OR inter-unit# OR interdepartment* or inter-department*)) OR AB (transfer* N2 (interfacilit* OR inter-facilit* OR intrafacilit* OR intra-facilit* OR interhospital* OR inter-hospital* OR intrahospital* OR intra-hospital* OR interward# OR inter-ward# OR interunit# OR inter-unit# OR interdepartment* or inter-department*)) | Expanders - Apply equivalent subjects Search modes - Proximity | 1,123 |
| S78 | TI (transfer* N2 (facilit* OR hospital* OR ward# OR unit# OR department* OR "level# of care" OR setting#)) OR AB (transfer* N2 (facilit* OR hospital* OR ward# OR unit# OR department* OR "level# of care" OR setting#)) | Expanders - Apply equivalent subjects Search modes - Proximity | 5,159 |
| S79 | TI (transition* N2 patient*) OR AB (transition* N2 patient*) | Expanders - Apply equivalent subjects Search modes - Proximity | 3,219 |
| S80 | TI (transition* N2 (care OR healthcare)) OR AB (transition* N2 (care OR healthcare)) | Expanders - Apply equivalent subjects Search modes - Proximity | 8,575 |
| S81 | TI (transition* N2 (interfacilit* OR inter-facilit* OR intrafacilit* OR intra-facilit* OR interhospital* OR inter-hospital* OR intrahospital* OR intra-hospital* OR interward# OR inter-ward# OR interunit# OR inter-unit# OR interdepartment* or inter-department*)) OR AB (transition* N2 (interfacilit* OR inter-facilit* OR intrafacilit* OR intra-facilit* OR interhospital* OR inter-hospital* OR intrahospital* OR intra-hospital* OR interward# OR inter-ward# OR interunit# OR inter-unit# OR interdepartment* or inter-department*)) | Expanders - Apply equivalent subjects Search modes - Proximity | 15 |
| S82 | TI (transition* N2 (facilit* OR hospital* OR ward# OR unit# OR department* OR "level# of care" OR setting#)) OR AB (transition* N2 (facilit* OR hospital* OR ward# OR unit# OR department* OR "level# of care" OR setting#)) | Expanders - Apply equivalent subjects Search modes - Proximity | 4,311 |
| S83 | TI (("transfer* to" OR "transfer* into") N2 (rehab* OR neurorehab* OR physiatr* OR "physical medicine" OR "complex continuing care" OR "skilled nursing" OR "extended care facilit*")) OR AB (("transfer* to" OR "transfer* into") N2 (rehab* OR neurorehab* OR physiatr* OR "physical medicine" OR "complex continuing care" OR "skilled nursing" OR "extended care facilit*")) | Expanders - Apply equivalent subjects Search modes - Proximity | 325 |
| S84 | TI ("transfer* for" W2 (rehab* OR neurorehab* OR physiatr* OR "physical medicine" OR "complex continuing care" OR "skilled nursing" OR "extended care facilit*")) OR AB ("transfer* for" W2 (rehab* OR neurorehab* OR physiatr* OR "physical medicine" OR "complex continuing care" OR "skilled nursing" OR "extended care facilit*")) | Expanders - Apply equivalent subjects Search modes - Proximity | 443 |
| S85 | TI (transfer* N2 ((to OR into) N1 (rehab* OR neurorehab* OR physiatr* OR "physical medicine" OR "complex continuing care" OR "skilled nursing" OR "extended care facilit*"))) OR AB (transfer* N2 ((to OR into) N1 (rehab* OR neurorehab* OR physiatr* OR "physical medicine" OR "complex continuing care" OR "skilled nursing" OR "extended care facilit*"))) | Expanders - Apply equivalent subjects Search modes - Proximity | 294 |
| S86 | TI (transfer* W2 (for W1 (rehab* OR neurorehab* OR physiatr* OR "physical medicine" OR "complex continuing care" OR "skilled nursing" OR "extended care facilit*"))) OR AB (transfer* W2 (for W1 (rehab* OR neurorehab* OR physiatr* OR "physical medicine" OR "complex continuing care" OR "skilled nursing" OR "extended care facilit*"))) | Expanders - Apply equivalent subjects Search modes - Proximity | 517 |
| S87 | TI (("discharg* to" OR "discharg* into") N2 (rehab* OR neurorehab* OR physiatr* OR "physical medicine" OR "complex continuing care" OR "skilled nursing" OR "extended care facilit*")) OR AB (("discharg* to" OR "discharg* into") N2 (rehab* OR neurorehab* OR physiatr* OR "physical medicine" OR "complex continuing care" OR "skilled nursing" OR "extended care facilit*")) | Expanders - Apply equivalent subjects Search modes - Proximity | 1,038 |
| S88 | TI ("discharg* for" W2 (rehab* OR neurorehab* OR physiatr* OR "physical medicine" OR "complex continuing care" OR "skilled nursing" OR "extended care facilit*")) OR AB ("discharg* for" W2 (rehab* OR neurorehab* OR physiatr* OR "physical medicine" OR "complex continuing care" OR "skilled nursing" OR "extended care facilit*")) | Expanders - Apply equivalent subjects Search modes - Proximity | 2,641 |
| S89 | TI (discharg* N2 ((to OR into) N1 (rehab* OR neurorehab* OR physiatr* OR "physical medicine" OR "complex continuing care" OR "skilled nursing" OR "extended care facilit*"))) OR AB (discharg* N2 ((to OR into) N1 (rehab* OR neurorehab* OR physiatr* OR "physical medicine" OR "complex continuing care" OR "skilled nursing" OR "extended care facilit*"))) | Expanders - Apply equivalent subjects Search modes - Proximity | 1,135 |
| S90 | TI (discharg* W2 (for W1 (rehab* OR neurorehab* OR physiatr* OR "physical medicine" OR "complex continuing care" OR "skilled nursing" OR "extended care facilit*"))) OR AB (discharg* W2 (for W1 (rehab* OR neurorehab* OR physiatr* OR "physical medicine" OR "complex continuing care" OR "skilled nursing" OR "extended care facilit*"))) | Expanders - Apply equivalent subjects Search modes - Proximity | 3,018 |
| S91 | TI ((“transition* to” OR “transition* into”) N2 (rehab* OR neurorehab* OR physiatr* OR "physical medicine" OR "complex continuing care" OR "skilled nursing" OR "extended care facilit*")) OR AB ((“transition* to” OR “transition* into”) N2 (rehab* OR neurorehab* OR physiatr* OR "physical medicine" OR "complex continuing care" OR "skilled nursing" OR "extended care facilit*")) | Expanders - Apply equivalent subjects Search modes - Proximity | 63 |
| S92 | TI ((“transition* for”) W2 (rehab* OR neurorehab* OR physiatr* OR "physical medicine" OR "complex continuing care" OR "skilled nursing" OR "extended care facilit*")) OR AB ((“transition* for”) W2 (rehab* OR neurorehab* OR physiatr* OR "physical medicine" OR "complex continuing care" OR "skilled nursing" OR "extended care facilit*")) | Expanders - Apply equivalent subjects Search modes - Proximity | 279 |
| S93 | TI (transition* N2 ((to OR into) N1 (rehab* OR neurorehab* OR physiatr* OR "physical medicine" OR "complex continuing care" OR "skilled nursing" OR "extended care facilit*"))) OR AB (transition* N2 ((to OR into) N1 (rehab* OR neurorehab* OR physiatr* OR "physical medicine" OR "complex continuing care" OR "skilled nursing" OR "extended care facilit*"))) | Expanders - Apply equivalent subjects Search modes - Proximity | 153 |
| S94 | TI (transition* W2 (for W1 (rehab* OR neurorehab* OR physiatr* OR "physical medicine" OR "complex continuing care" OR "skilled nursing" OR "extended care facilit*"))) OR AB (transition* W2 (for W1 (rehab* OR neurorehab* OR physiatr* OR "physical medicine" OR "complex continuing care" OR "skilled nursing" OR "extended care facilit*"))) | Expanders - Apply equivalent subjects Search modes - Proximity | 344 |
| S95 | S71 OR S72 OR S73 OR S74 OR S75 OR S76 OR S77 OR S78 OR S79 OR S80 OR S81 OR S82 OR S83 OR S84 OR S85 OR S86 OR S87 OR S88 OR S89 OR S90 OR S91 OR S92 OR S93 OR S94 | Expanders - Apply equivalent subjects Search modes - Proximity | 41,566 |
| S96 | (MH "Communication+") | Expanders - Apply equivalent subjects Search modes - Proximity | 356,422 |
| S97 | (MH "Documentation") | Expanders - Apply equivalent subjects Search modes - Proximity | 35,864 |
| S98 | (MH "Charting+") | Expanders - Apply equivalent subjects Search modes - Proximity | 3,448 |
| S99 | TI (communicat*) OR AB (communicat*) | Expanders - Apply equivalent subjects Search modes - Proximity | 161,384 |
| S100 | TI (document*) OR AB (document*) | Expanders - Apply equivalent subjects Search modes - Proximity | 143,833 |
| S101 | TI (information* N3 (exchang* OR share# OR sharing OR transfer* OR continuit* OR continuum*)) OR AB (information* N3 (exchang* OR share# OR sharing OR transfer* OR continuit* OR continuum*)) | Expanders - Apply equivalent subjects Search modes - Proximity | 13,104 |
| S102 | S96 OR S97 OR S98 OR S99 OR S100 OR S101 | Expanders - Apply equivalent subjects Search modes - Proximity | 610,946 |
| S103 | S95 AND S102 | Expanders - Apply equivalent subjects Search modes - Proximity | 7,113 |
| S104 | S70 OR S103 | Expanders - Apply equivalent subjects Search modes - Proximity | 21,865 |
| S105 | S38 AND S104 | Expanders - Apply equivalent subjects Search modes - Proximity | 1,102 |

## Web of Science

**Database:** Web of Science Core Collection

| **#** | **Search Query** | **Results** |
| --- | --- | --- |
| 1 | TS=((rehab* or neurorehab* or physiatr* or "physical medicine") NEAR/2 hospital*) | 8190 |
| 2 | TS=((rehab* or neurorehab* or physiatr* or "physical medicine") NEAR/2 (inhospital* or "in-hospital*")) | 789 |
| 3 | TS=((rehab* or neurorehab* or physiatr* or "physical medicine") NEAR/2 (center* or centre*)) | 11159 |
| 4 | TS=((rehab* or neurorehab* or physiatr* or "physical medicine") NEAR/2 facilit*) | 6070 |
| 5 | TS= ((rehab* or neurorehab* or physiatr* or "physical medicine") NEAR/2 (inpatient* or "in-patient*")) | 15374 |
| 6 | TS=((rehab* or neurorehab* or physiatr* or "physical medicine") NEAR/2 setting*) | 7524 |
| 7 | TS=((rehab* or neurorehab* or physiatr* or "physical medicine") NEAR/2 (ward or wards)) | 1370 |
| 8 | TS=((rehab* or neurorehab* or physiatr* or "physical medicine") NEAR/2 (unit or units)) | 4789 |
| 9 | TS=((rehab* or neurorehab* or physiatr* or "physical medicine") NEAR/2 department*) | 2143 |
| 10 | TS=((rehab* or neurorehab* or physiatr* or "physical medicine") NEAR/2 (admit* or admission*)) | 3433 |
| 11 | TS=((rehab* or neurorehab* or physiatr* or "physical medicine") NEAR/2 program*) | 30479 |
| 12 | TS=((rehab* or neurorehab* or physiatr* or "physical medicine") NEAR/2 institut*) | 1495 |
| 13 | TS=((rehab* or neurorehab* or physiatr* or "physical medicine") NEAR/2 floor$) | 477 |
| 14 | TS=((rehab* or neurorehab* or physiatr* or "physical medicine") NEAR/2 section*) | 392 |
| 15 | TS=((rehab* or neurorehab* or physiatr* or "physical medicine") NEAR/2 (bed or beds or bedded)) | 308 |
| 16 | TS=((rehab* or neurorehab* or physiatr* or "physical medicine") NEAR/2 (stay or stays or stayed or staying)) | 1421 |
| 17 | TS=((rehab* or neurorehab* or physiatr* or "physical medicine") NEAR/2 resident$) | 309 |
| 18 | TS=((rehab* or neurorehab* or physiatr* or "physical medicine") NEAR/2 acute*) | 4909 |
| 19 | TS=((rehab* or neurorehab* or physiatr* or "physical medicine") NEAR/2 (post-acute* or postacute*)) | 1237 |
| 20 | TS=((rehab* or neurorehab* or physiatr* or "physical medicine") NEAR/2 (sub-acute* or subacute*)) | 834 |
| 21 | TS=(("transfer* to" or "transfer* into" or "transfer* for") NEAR/2 (rehab* or neurorehab* or physiatr* or "physical medicine")) | 670 |
| 22 | TS=(transfer* NEAR/2 ((to or into or for) NEAR/1 (rehab* or neurorehab* or physiatr* or "physical medicine"))) | 640 |
| 23 | TS=(("discharg* to" or "discharg* into" or "discharg* for") NEAR/2 (rehab* or neurorehab* or physiatr* or "physical medicine")) | 1421 |
| 24 | TS=(discharg* NEAR/2 ((to or into or for) NEAR/1 (rehab* or neurorehab* or physiatr* or "physical medicine"))) | 1673 |
| 25 | TS=(("transition* to" or "transition* into" or "transition* for") NEAR/2 (rehab* or neurorehab* or physiatr* or "physical medicine")) | 71 |
| 26 | TS=(transition* NEAR/2 ((to or into or for) NEAR/1 (rehab* or neurorehab* or physiatr* or "physical medicine"))) | 170 |
| 27 | TS=("skilled nursing") | 4746 |
| 28 | TS=("extended care" NEAR/2 facilit*) | 388 |
| 29 | TS=("complex continuing care") | 96 |
| 30 | #1 OR #2 OR #3 OR #4 OR #5 OR #6 OR #7 OR #8 OR #9 OR #10 OR #11 OR #12 OR #13 OR #14 OR #15 OR #16 OR #17 OR #18 OR #19 OR #20 OR #21 OR #22 OR #23 OR #24 OR #25 OR #26 OR #27 OR #28 OR #29 | 79748 |
| 31 | TS=(handoff*) | 9865 |
| 32 | TS=("hand$ off*" or "handing off" or "handed off") | 3634 |
| 33 | TS=(handover*) | 15181 |
| 34 | TS=("hand$ over*" or "handing over" or "handed over") | 5413 |
| 35 | TS=(signout*) | 110 |
| 36 | TS=("sign$ out*" or "signing out" or "signed out") | 1200 |
| 37 | TS=(signover*) | 1 |
| 38 | TS=("sign$ over*" or "signing over" or "signed over") | 658 |
| 39 | TS=("transfer* of" NEAR/2 care) | 1013 |
| 40 | TS=(transfer* NEAR/0 care) | 623 |
| 41 | TS=("transfer* of" NEAR/2 healthcare) | 66 |
| 42 | TS=(transfer* NEAR/0 healthcare) | 37 |
| 43 | TS=("transfer* in" NEAR/2 care) | 181 |
| 44 | TS=("transfer* in" NEAR/2 healthcare) | 29 |
| 45 | TS=(transfer* NEAR/2 (accountabilit* or responsibilit* or authorit* or decision-making)) | 2244 |
| 46 | TS=("shift report*" or shift-to-shift or intershift$ or inter-shift$ or end-of-shift) | 791 |
| 47 | TS=((bedside* or “bed side*”) NEAR/2 report*) | 304 |
| 48 | TS=(physician$-to-physician* or doctor$-to-doctor* or hospitalist$-to-hospitalist* or surgeon$-to-surgeon* or resident$-to-resident*) | 406 |
| 49 | TS=(physician$-to-physiatrist* or doctor$-to-physiatrist* or hospitalist$-to-physiatrist* or surgeon$-to-physiatrist* or resident$-to-physiatrist* or physician$-to-doctor* or physician$-to-hospitalist* or physician$-to-surgeon* or physician$-to-resident* or doctor$-to-physician* or doctor$-to-hospitalist* or doctor$-to-surgeon* or doctor$-to-resident* or hospitalist$-to-physician* or hospitalist$-to-doctor* or hospitalist$-to-surgeon* or hospitalist$-to-resident* or surgeon$-to-physician* or surgeon$-to-doctor* or surgeon$-to-hospitalist* or surgeon$-to-resident* or resident$-to-physician* or resident$-to-doctor* or resident$-to-hospitalist* or resident$-to-surgeon*) | 24 |
| 50 | TS=(provider$-to-provider* or clinician$-to-clinician*) | 184 |
| 51 | TS=((physician$-physician* or doctor$-doctor* or hospitalist$-hospitalist* or surgeon$-surgeon* or resident$-resident*) NEAR/2 (communicat* or document* or transfer* or record* or report* or note$ or brief* or consult* or share$ or sharing)) | 105 |
| 52 | TS=((physician$-physiatrist* or doctor$-physiatrist* or hospitalist$-physiatrist* or surgeon$-physiatrist* or resident$-physiatrist* or physician$-doctor* or physician$-hospitalist* or physician$-surgeon* or physician$-resident* or doctor$-physician* or doctor$-hospitalist* or doctor$-surgeon* or doctor$-resident* or hospitalist$-physician* or hospitalist$-doctor* or hospitalist$-surgeon* or hospitalist$-resident* or surgeon$-physician* or surgeon$-doctor* or surgeon$-hospitalist* or surgeon$-resident* or resident$-physician* or resident$-doctor* or resident$-hospitalist* or resident$-surgeon*) NEAR/2 (communicat* or document* or transfer* or record* or report* or note$ or brief* or consult* or share$ or sharing)) | 255 |
| 53 | TS=((provider$-provider* or clinician$-clinician*) NEAR/2 (communicat* or document* or transfer* or record* or report* or note$ or brief* or consult* or share$ or sharing)) | 89 |
| 54 | TS=(transfer* NEAR/1 (agreement* or note$ or record* or report* or document* or communicat* or consult*)) | 6670 |
| 55 | TS=(transfer* NEAR/1 (process* or procedure* or protocol* or checklist* or "check list*" or mnemonic*)) | 70884 |
| 56 | TS=(transfer* NEAR/1 (decision* or summar*)) | 1430 |
| 57 | TS=(safe* NEAR/2 (transfer* or transition*)) | 3958 |
| 58 | TS=("transition$ of" NEAR/1 (care or healthcare)) | 3502 |
| 59 | TS=("transition$ in" NEAR/1 (care or healthcare)) | 1043 |
| 60 | #31 OR #32 OR #33 OR #34 OR #35 OR #36 OR #37 OR #38 OR #39 OR #40 OR #41 OR #42 OR #43 OR #44 OR #45 OR #46 OR #47 OR #48 OR #49 OR #50 OR #51 OR #52 OR #53 OR #54 OR #55 OR #56 OR #57 OR #58 OR #59 | 124526 |
| 61 | TS=(transfer* NEAR/2 patient*) | 19161 |
| 62 | TS=(transfer* NEAR/2 (care or healthcare)) | 5315 |
| 63 | TS=(transfer* NEAR/2 (interfacilit* or inter-facilit* or intrafacilit* or intra-facilit* or interhospital* or inter-hospital* or intrahospital* or intra-hospital* or interward$ or inter-ward$ or interunit$ or inter-unit$ or interdepartment* or inter-department*)) | 2680 |
| 64 | TS=(transfer* NEAR/2 (facilit* or hospital* or ward$ or unit$ or department* or "level$ of care" or setting$)) | 36059 |
| 65 | TS=(transition* NEAR/2 patient*) | 7886 |
| 66 | TS=(transition* NEAR/2 (care or healthcare)) | 15252 |
| 67 | TS=(transition* NEAR/2 (interfacilit* or inter-facilit* or intrafacilit* or intra-facilit* or interhospital* or inter-hospital* or intrahospital* or intra-hospital* or interward$ or inter-ward$ or interunit$ or inter-unit$ or interdepartment* or inter-department*)) | 31 |
| 68 | TS=(transition* NEAR/2 (facilit* or hospital* or ward$ or unit$ or department* or "level$ of care" or setting$)) | 14664 |
| 69 | TS=(("transfer* to" or "transfer* into" or "transfer* for") NEAR/2 (rehab* or neurorehab* or physiatr* or "physical medicine" or "complex continuing care" or "skilled nursing" or "extended care facilit*")) | 734 |
| 70 | TS=(transfer* NEAR/2 ((to or into or for) NEAR/1 (rehab* or neurorehab* or physiatr* or "physical medicine" or "complex continuing care" or "skilled nursing" or "extended care facilit*"))) | 712 |
| 71 | TS=(("discharg* to" or "discharg* into" or "discharg* for") NEAR/2 (rehab* or neurorehab* or physiatr* or "physical medicine" or "complex continuing care" or "skilled nursing" or "extended care facilit*")) | 2212 |
| 72 | TS=(discharg* NEAR/2 ((to or into or for) NEAR/1 (rehab* or neurorehab* or physiatr* or "physical medicine" or "complex continuing care" or "skilled nursing" or "extended care facilit*"))) | 2493 |
| 73 | TS=(("transition* to" or "transition* into" or "transition* for") NEAR/2 (rehab* or neurorehab* or physiatr* or "physical medicine" or "complex continuing care" or "skilled nursing" or "extended care facilit*")) | 81 |
| 74 | TS=(transition* NEAR/2 ((to or into or for) NEAR/1 (rehab* or neurorehab* or physiatr* or "physical medicine" or "complex continuing care" or "skilled nursing" or "extended care facilit*"))) | 238 |
| 75 | #61 OR #62 OR #63 OR #64 OR #65 OR #66 OR #67 OR #68 OR #69 OR #70 OR #71 OR #72 OR #73 OR #74 | 91106 |
| 76 | TS=(communicat*) | 1690439 |
| 77 | TS=(document*) | 1032003 |
| 78 | TS=(information* NEAR/3 (exchang* or share$ or sharing or transfer* or continuit* or continuum*)) | 136761 |
| 79 | #76 OR #77 OR #78 | 2772967 |
| 80 | #75 AND #79 | 9668 |
| 81 | #60 OR #80 | 131614 |
| 82 | #30 AND #81 | 851 |
